# Identification of Novel Loci Associated With Hip Shape: A Meta‐Analysis of Genomewide Association Studies

**DOI:** 10.1002/jbmr.3605

**Published:** 2018-11-26

**Authors:** Denis A Baird, Daniel S Evans, Frederick K Kamanu, Jennifer S Gregory, Fiona R Saunders, Claudiu V Giuraniuc, Rebecca J Barr, Richard M Aspden, Deborah Jenkins, Douglas P Kiel, Eric S Orwoll, Steven R Cummings, Nancy E Lane, Benjamin H Mullin, Frances MK Williams, J Brent Richards, Scott G Wilson, Tim D Spector, Benjamin G Faber, Deborah A Lawlor, Elin Grundberg, Claes Ohlsson, Ulrika Pettersson‐Kymmer, Terence D Capellini, Daniel Richard, Thomas J Beck, David M Evans, Lavinia Paternoster, David Karasik, Jonathan H Tobias

**Affiliations:** ^1^ Musculoskeletal Research Unit University of Bristol Bristol UK; ^2^ California Pacific Medical Center Research Institute San Francisco CA USA; ^3^ Institute for Aging Research Hebrew SeniorLife Department of Medicine Beth Israel Deaconess Medical Center and Harvard Medical School Boston MA USA; ^4^ Arthritis and Musculoskeletal Medicine University of Aberdeen Aberdeen UK; ^5^ MEMO Research University of Dundee Dundee UK; ^6^ Broad Institute of MIT and Harvard Boston MA USA; ^7^ School of Medicine Oregon Health and Science University Portland OR USA; ^8^ University of California at Davis Sacramento CA USA; ^9^ Department of Endocrinology and Diabetes Sir Charles Gairdner Hospital Nedlands Australia; ^10^ School of Biomedical Sciences University of Western Australia Perth Australia; ^11^ Department of Twin Research and Genetic Epidemiology King's College London London UK; ^12^ Departments of Medicine, Human Genetics, Epidemiology, and Biostatistics Jewish General Hospital McGill University Montreal Canada; ^13^ MRC Integrative Epidemiology Unit University of Bristol Bristol UK; ^14^ Department of Human Genetics McGill University Montreal Canada; ^15^ Centre for Bone and Arthritis Research Institute of Medicine University of Gothenburg Gothenburg Sweden; ^16^ Department of Pharmacology and Clinical Neurosciences Umea University Umea Sweden; ^17^ Human Evolutionary Biology Harvard University Boston MA USA; ^18^ Beck Radiological Innovations Catonsville MD USA; ^19^ University of Queensland Diamantina Institute Translational Research Institute Brisbane Australia; ^20^ Azrieli Faculty of Medicine Bar Ilan University Safed Israel

**Keywords:** HIP SHAPE, OSTEOARTHRITIS, HIP FRACTURE RISK, DXA, GWAS

## Abstract

We aimed to report the first genomewide association study (GWAS) meta‐analysis of dual‐energy X‐ray absorptiometry (DXA)‐derived hip shape, which is thought to be related to the risk of both hip osteoarthritis and hip fracture. Ten hip shape modes (HSMs) were derived by statistical shape modeling using SHAPE software, from hip DXA scans in the Avon Longitudinal Study of Parents and Children (ALSPAC; adult females), TwinsUK (mixed sex), Framingham Osteoporosis Study (FOS; mixed), Osteoporotic Fractures in Men study (MrOS), and Study of Osteoporotic Fractures (SOF; females) (total *N* = 15,934). Associations were adjusted for age, sex, and ancestry. Five genomewide significant (*p* < 5 × 10^−9^, adjusted for 10 independent outcomes) single‐nucleotide polymorphisms (SNPs) were associated with HSM1, and three SNPs with HSM2. One SNP, in high linkage disequilibrium with rs2158915 associated with HSM1, was associated with HSM5 at genomewide significance. In a look‐up of previous GWASs, three of the identified SNPs were associated with hip osteoarthritis, one with hip fracture, and five with height. Seven SNPs were within 200 kb of genes involved in endochondral bone formation, namely *SOX9*, *PTHrP*, *RUNX1*, *NKX3‐2*, *FGFR4*, *DICER1*, and *HHIP*. The SNP adjacent to *DICER1* also showed osteoblast cis‐regulatory activity of *GSC*, in which mutations have previously been reported to cause hip dysplasia. For three of the lead SNPs, SNPs in high LD (*r* ^2^ > 0.5) were identified, which intersected with open chromatin sites as detected by ATAC‐seq performed on embryonic mouse proximal femora. In conclusion, we identified eight SNPs independently associated with hip shape, most of which were associated with height and/or mapped close to endochondral bone formation genes, consistent with a contribution of processes involved in limb growth to hip shape and pathological sequelae. These findings raise the possibility that genetic studies of hip shape might help in understanding potential pathways involved in hip osteoarthritis and hip fracture. © 2018 The Authors. *Journal of Bone and Mineral Research* Published by Wiley Periodicals, Inc.

## Introduction

Alterations in hip shape have important implications for disease risk. For example, cam‐type deformity caused by extra bone growth around the anterolateral aspect of the femoral head leading to femoro‐acetabular impingement (FAI) is associated with premature onset of hip osteoarthritis (OA).^1^ In the Rotterdam study, individuals with cam deformity and acetabular dysplasia had a twofold increased risk of radiographic hip OA compared with controls.^2^ Hip shape has also been suggested to predict subsequent hip fracture risk.^3^

Statistical shape modeling (SSM) has been used to describe overall hip shape, using principal component analysis (PCA) to derive a set of orthogonal hip shape modes (HSMs).^4^ This method has been applied to investigate genetic influences on hip OA acting through alterations in hip shape, using a candidate gene approach. For example, two independent SNPs within *FRZB* were previously reported to be associated with radiographically defined hip shape in a nested case‐control study of older women from Study of Osteoporotic Fractures (SOF) (*n* = 1046),^5^ as were SNPs within *ASTN2* and *GLT8D1* and close to *IFRD1* in 929 subjects with unilateral radiographic hip OA.^6^ HSMs derived from SSMs show evidence of being heritable (eg, mode 1 heritability estimated as 0.23^7^), justifying the search for genetic influences.

SSM has recently been applied to DXA scans to explore the role of hip shape in the incidence and progression of hip OA, using a similar approach to radiograph‐based analyses described above, with the exception that shape models also included the acetabular roof.^8^, ^9^ In the present study, we performed the first GWAS meta‐analysis to identify novel genetic factors associated with hip shape, based on measures derived from hip DXA scans by SSM, after combining scans from five distinct population‐based cohorts to ensure a sufficiently large sample (*n* = 15,934) for genetic discovery.

## Materials and Methods

### Participating cohorts

Hip DXA scans were obtained from the Study of Osteoporotic Fractures in Men (MrOS; first images taken), the Study of Osteoporotic Fractures (SOF; first images taken), Framingham Osteoporosis Study (FOS; first images taken), TwinsUK, and Avon Longitudinal Study of Parents and Children (ALSPAC; mothers’ first images taken). See Supplemental Methods for more details.

### Statistical shape modeling

Hip DXA scans were uploaded to SHAPE software (University of Aberdeen). One hip DXA scan per individual was used, the left side being selected in preference. SHAPE automatically placed 53 predefined points on the upper femur and adjacent acetabulum, related to key anatomical positions.^9^ Point placement was checked and manually realigned if required by a trained operator to ensure accurate positioning on the cortical outline. Hip shape size and rotation were standardized by Procrustes analysis. PCA was then performed on the point coordinates from the combined sample of scans collected across all five cohorts, producing a set of HSMs that describe linearly independent variations in hip shape. The first 10 modes, accounting for 85% of total variance, were used as outcomes (Supplemental Fig. S1). HSMs are expressed as deviation from the mean shape in the combined hip shape sample in standard normal units (mean = 0, SD = 1). Outliers (mode scores above or below 4 SDs) were manually checked by two operators, point placement corrected where necessary, SSM repeated, and HSMs then passed to participating cohorts for combining with genetic data.

The distribution of the HSMs within the cohorts was examined and confirmed to follow a standard normal distribution, with the exception of HSM1, which was skewed due to scanner differences between cohorts (Supplemental Table S1) (GE Lunar [Madison, WI, USA] scanners were used in ALSPAC and FOS, and Hologic [Waltham, MA, USA] scanners in MrOs, SOF and TwinsUK [Supplemental Methods]). Therefore, the genetic effect estimate (and SE) within each cohort was rescaled by 1/SD before conducting the meta‐analysis to provide a standardized comparison across the modes. For each genomewide significant SNP, SHAPE was used to plot the overall effect on hip shape, using a linear combination of beta estimates for all nominally significant SNP‐HSM associations. Because the influences of common genetic variants on hip shape were too small to visualize, beta coefficients were multiplied 20‐fold for illustrative purposes.

### Genomewide association study and meta‐analysis

For each individual cohort, a GWAS was performed for the top 10 HSM, using genotypes imputed from Haplotype Reference Consortium panel version 1 (ALSPAC, FOS, TwinsUK) or 1000 Genomes Project phase 1 version 3 (MrOS, SOF). Analyses were adjusted for age at scan and population substructure using ancestry‐derived PCs (and sex for FOS and TwinsUK). Sex was not adjusted for in the all‐male MrOS cohort or the all‐female cohort SOF. Additional height‐adjusted GWASs for HSM1 and HSM2 were run as sensitivity analyses. GWAS was performed in ALSPAC using SNPtest, in MrOS and SOF using PLINK, and in TwinsUK using GEMMA (genomewide efficient mixed model association)^10^ to control for familial relatedness within a cohort. GWAS in FOS used linear mixed‐effects models to account for the familial relationships (the R Kinship2 package; https://cran.r-project.org/web/packages/available_packages_by_name.html). A fixed effects meta‐analysis was conducted for common SNPs (MAF > 1%) across the five studies, using the R package EasyQC.^11^ Info score < 0.4 and *r* ^2^_hat < 0.3 were used to exclude poorly imputed SNPs. The genomic inflation factor (λ) was used to check for *p* value inflation on QQ plots. Manhattan plots were generated in the R package EasyStrata^12^ and regional association plots using LocusZoom.^13^ Forest plots were generated using the R package ggplot2. A random effects meta‐analysis was also run to generate *I* ^2^ statistics to test for heterogeneity using the GWAMA package.^14^ A Bonferroni‐corrected genomewide significance threshold of *p* < 5 × 10^−9^ was used to account for testing genomewide SNPs across 10 outcomes (HSMs).

### SNP heritability analysis

SNP heritabilities of the HSMs were computed on GWAS summary statistics by LD score regression, using the LDSC module.^15^ LD score regression was also used to examine genetic correlations between the HSMs and hip osteoarthritis,^16^ anthropometric traits (height^17^ and waist circumference^18^), and femoral neck bone mineral density (FN BMD).^19^

### SNP associations with other traits

GWAS‐identified SNPs were examined in relation to OA using summary statistics available from the arcOGEN hip OA GWAS^16^ (https://www.arcogen.org.uk/), a recent UK Biobank hip OA GWAS,^20^ and a hip fracture GWAS (as yet unpublished; see Supplemental Methods). In addition, a look‐up was performed on GWASs of anthropometric and bone‐related traits collected in the MRBase database.^21^ Approximate Bayesian colocalization analysis was performed to determine the posterior probability (PP) that these associations share the same causal SNP within the genomic region. The “coloc.abf” function in the coloc package in R was used,^9^ using the default setting for the priors (*p* = 1 × 10^−4^ for each trait to be associated separately and *p* = 1 × 10^−5^ for both traits to be associated jointly).

### Functional analyses

To further whittle down GWAS loci to fewer, potentially causative variants, several computational and wet‐lab experimental analyses were performed. First, the function of genes in the genomic region of the lead SNPs was explored, including known skeletal functions, as well as whether Mendelian diseases (curated from Online Mendelian Inheritance in Man [OMIM]) that have been associated with a skeletal abnormality, and/or whether mouse knockouts have been reported demonstrating a skeletal phenotype. Second, to identify possible roles of lead and proxy variants in cis‐regulation, GWAS‐identified SNPs were examined in an osteoblast eQTL genomewide data set, based on 95 unrelated donors.^22^ For the hip shape‐associated SNP(s) showing evidence of cis‐regulatory activity, evidence of a direct signal between the eQTL and hip shape (rather than through coincidental sharing due to linkage) was sought by heterogeneity of association across multiple SNPs in the cis‐eQTL region using HEIDI test statistics.^23^ Third, whether lead and/or proxy SNPs represent sites of transcriptional regulation was finally evaluated using a combination of bioinformatics tools including RegulomeDB and with data acquired from an experimental approach, the Assay for Transposase‐Accessible Chromatin followed by sequencing (ATAC‐seq)^24^ applied to mouse proximal femur. See Supplemental Methods for more details.

## Results

### Hip shape GWAS

Hip shape measures were available in 19,379 individuals across the five participating cohorts (Supplemental Table S2), in whom genotype data were available in 15,934. A total of 7,191,926 SNPs across the five cohorts passed quality‐control filters. Inspection of the QQ plots, both within the GWASs conducted on individual cohorts and for the overall meta‐analysis, showed no inflation of *p* values (genomic inflation factors for all HSM GWASs ≤ 1.07). Four of the 10 HSMs were estimated to have a small SNPwise heritable component by LD score regression (HSM1: *h* ^2^ = 0.072 [95% CI 0.0067, 0.14]; HSM2: 0.12 [0.041,0.19]; HSM5: 0.075 [0.012, 0.14]; HSM8: 0.12 [0.040, 0.16]). LD score regression demonstrated a moderate genetic correlation between HSM2 and height (*r*_g _= 0.16 [0.031, 0.29], *p* = 0.015) and body mass index (BMI)‐adjusted waist circumference (an independent predictor of mortality^25^) (*r*_g _= 0.31 [0.14, 0.48], *p* = 0.0002).

**Figure 1:**
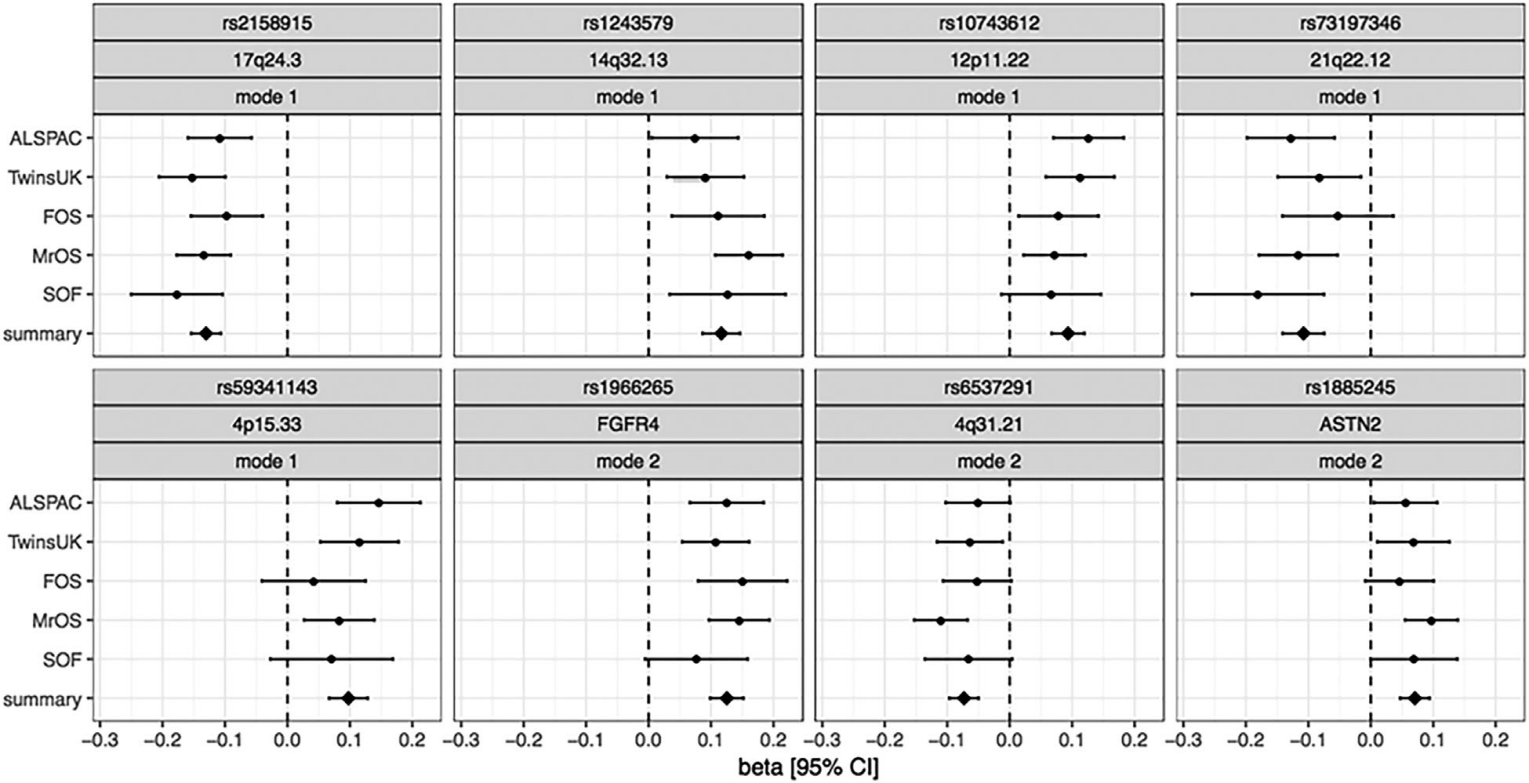
Forest plot showing association between lead SNPs shown in Table 1 and HSM1 or HSM2, by cohort. Subsequent random effects meta‐analysis showed little evidence of heterogeneity as reflected by low *I* ^2^ (rs215895 = 0.06, rs1243579 = 0.15, rs10473612 = 0, rs73197346 = 0.06, rs59341143 = 0.13, rs1966265 = 0, rs1542725 = 0.08, rs1885245 = 0).

**Figure 2:**
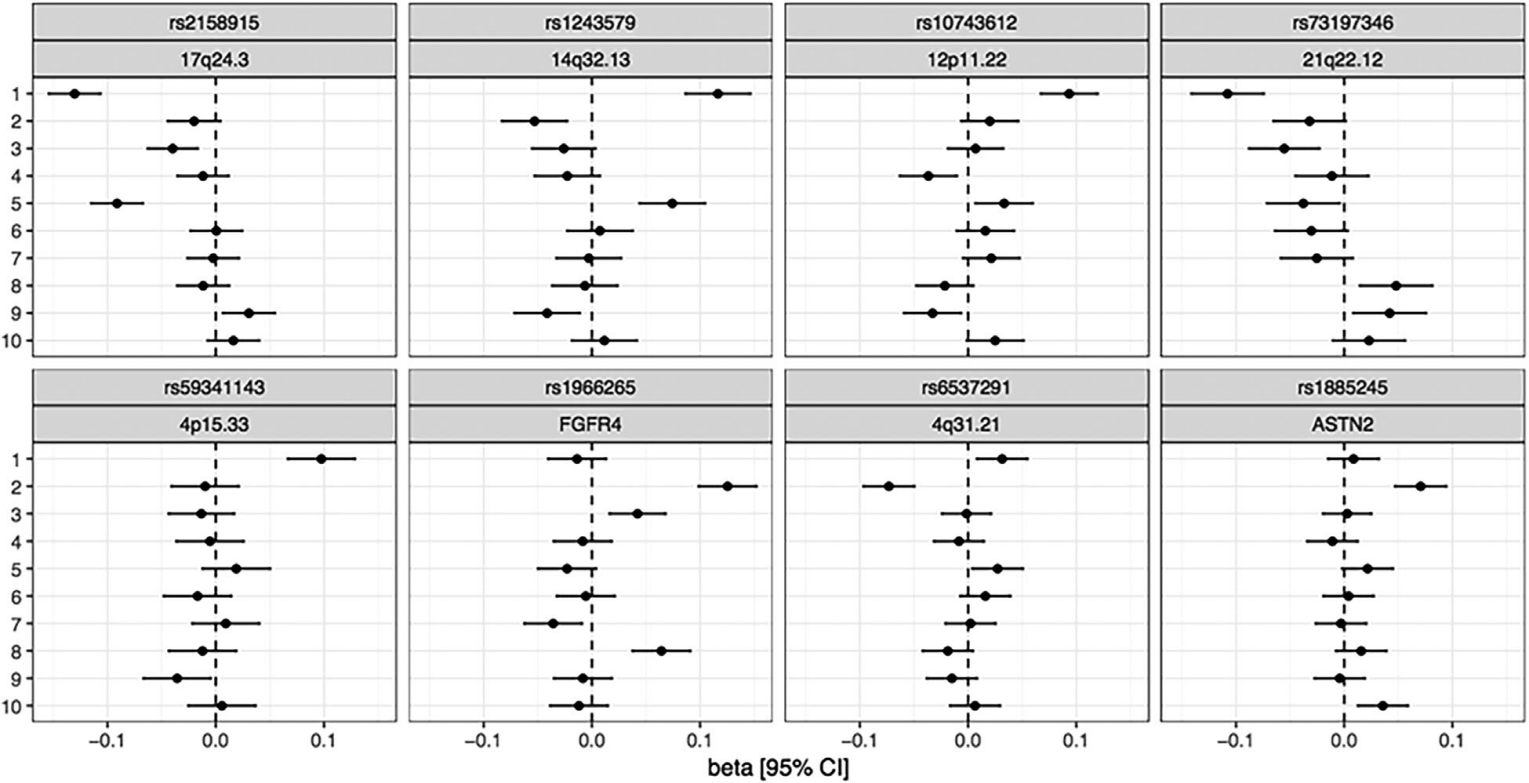
Association between lead SNPs shown in Table 1 and each HSM. Results show effect size (SD) with 95% confidence interval.

**Figure 3:**
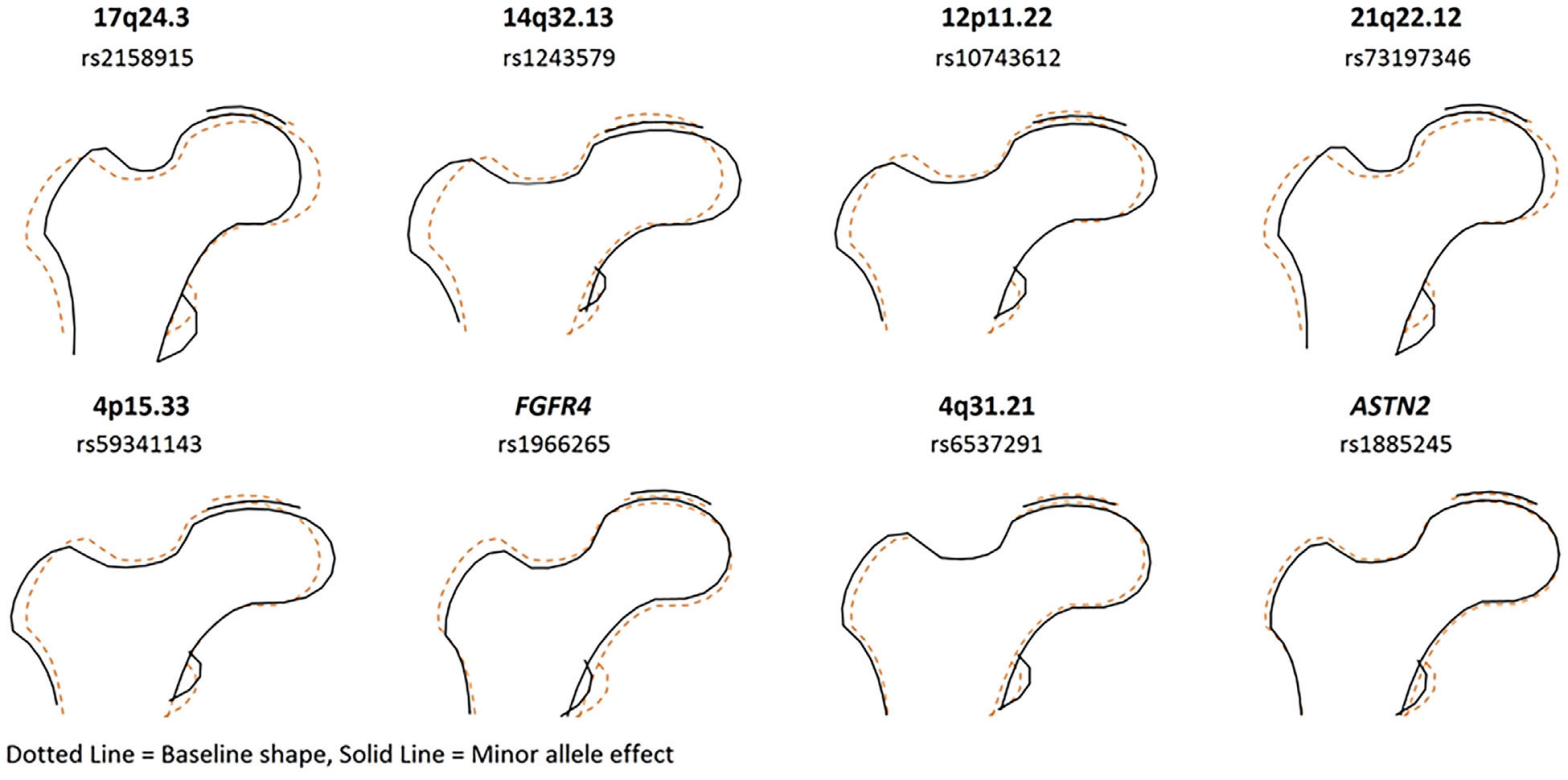
Effect of SNPs shown in Table 1 on hip shape. The overall effect of each SNP shown in Table 1 on hip shape was subsequently modeled for the minor (ie, effect) allele, by entering the beta value for each association of that SNP with HSMs at *p* < 0.05, into SHAPE (see Fig. 2; beta estimates multiplied by 20 for illustrative purposes).

Manhattan plots indicated genomewide significant associations for HSM1, HSM2, and HSM5 (Supplemental Fig. S3). Nine SNPs were associated with hip shape in the meta‐analysis at Bonferroni‐corrected genomewide significance (*p* < 5 × 10^−9^), five for HSM1, three for HSM2, and one for HSM5 (Table 1). Genetic associations showed little evidence of heterogeneity between cohorts as reflected by low *I* ^2^ estimates (Fig. 1). SNPs associated with one HSM at genomewide significance were also associated with other HSMs at nominal significance threshold (*p* < 0.05) (Fig. 2). On illustrating the overall effect of each genomewide significant SNP on hip shape, based on a 20‐fold magnification, the minor allele was associated with the following shape differences: altered aspect ratio (ie, width relative to height) of the upper femur (wider: rs1243579, rs10473612, rs59341143, and rs6537291; narrower: rs2158915 and rs73197346); narrower and more angulated femoral neck (rs1966265); smaller femoral head (rs1885245) (Fig. 3).

**Table 1:** Genetic Associations Detected in the Meta‐Analysis (*p* < 5 × 10^−9^)

HSM	SNP	Gene/locus	EA	EAF	Beta	SE	*p*
1	rs2158915	17q24.3	G	0.35	–0.13	0.012	8.47 × 10^−27^
1	rs1243579	14q32.13	G	0.15	0.12	0.015	2.85 × 10^−14^
1	rs10743612	12p11.22	A	0.24	0.093	0.013	2.91 × 10^−12^
1	rs73197346	21q22.12	C	0.14	–0.11	0.017	2.52 × 10^−10^
1	rs59341143	4p15.33	C	0.15	0.098	0.016	6.53 × 10^−10^
2	rs1966265	FGFR4	T	0.38	0.13	0.014	3.73 × 10^−20^
2	rs6537291	4q31.21	A	0.38	–0.073	0.012	1.01 × 10^−9^
2	rs1885245	ASTN2	G	0.40	0.071	0.012	4.95 × 10^−9^
5	rs2160442	17q24.3	A	0.35	–0.092	0.012	5.18 × 10^−14^

Nine single‐nucleotide polymorphisms (SNPs) exceeding cut‐off for genomewide significance for hip shape modes (HSMs), describing the SNP identifier, gene (locus where intergenic), effect (ie, minor) allele (EA), effect allele frequency (EAF), effect estimate (beta), and *p* value (*p*).

#### HSM1 and HSM5

The strongest association with HSM1 was for rs2158915, an intergenic SNP at the 17q24.3 locus upstream of *SOX9*, with the minor allele inversely related to HSM1 (*p* = 8.47 × 10^−27^; Supplemental Fig. S4), indicating a narrower aspect ratio of the upper femur (Supplemental Fig. S1). The same locus was associated with HSM5 in this study, with which rs2160442 (in perfect linkage with rs2158915) showed the strongest association (*p* = 5.18 × 10^−14^). HSM1 and HSM5 showed similar patterns of associations across all the SNPs within this region (Supplemental Fig. S4), co‐localization analysis strongly favoring the hypothesis that these modes share a common genetic signal (posterior probability [PP] of shared causal variant = 98.4%). Four additional SNPs were associated with HSM1 at a genomewide significance level: rs1243579, an intergenic SNP between *GSC* and *DICER1* (*p* = 2.85 × 10^−14^; 14q32.13; Supplemental Fig. S5); rs10743612, an intergenic SNP between *KLHL42* and *PTHLH* (*p* = 2.91 × 10^−12^; 12p11.22; Supplemental Fig. S6); rs73197346, an intergenic SNP between *RUNX1* and *MIR802* (*p* = 2.52 × 10^−10^; 21q22.12; Supplemental Fig. S7); rs59341143, an intergenic SNP between *RAB28* and *NKX3‐2* (*p* = 6.53 × 10^−10^; 4p15.33; Supplemental Fig. S8). Two suggestive associations were also detected for HSM1: rs6458443, an intronic SNP in *RUNX2* (*p* = 6.69 × 10^−9^), and rs6564537, an intronic SNP in *WWOX* (*p* = 1.08 × 10^−8^) (Supplemental Table S3).

#### HSM2

The minor allele of rs1966265, a missense SNP of *FGFR4*, was positively associated with HSM2, indicating a narrower femoral neck (Supplemental Fig. S1) (*p* = 3.73 × 10^−20^; Supplemental Fig. S9). PolyPhen2 revealed that rs1966265 is unlikely to affect protein function, implying that rs1966265 may not be the causal SNP at this locus. For example, rs12519145 in high LD with rs1966265 showed strong evidence of enhancer activity on RegulomeDB and Haploreg annotation and would be a stronger functional candidate (Supplemental Table S5). Two additional SNPs were associated with HSM2 at a genomewide significance level: rs6537291, an intergenic SNP upstream to *HHIP* (*p* = 1.01 × 10^−9^; 4q31.21; Supplemental Fig. S10); rs1885245, an intronic SNP of *ASTN2* (*p* = 4.95 × 10^−9^; Supplemental Fig. S11). In addition, rs17725170, an intergenic SNP between *IRX1* and *ADAMTS16*, showed a suggestive association with HSM2 (*p* = 5.69 × 10^−9^; 5p15.33; Supplemental Table S3).

### Variants shared with other traits

#### Height

The minor allele of the *FGFR4* SNP was related to greater stature (*p* = 3.8 × 10^−16^) (Table 2); co‐localization analysis strongly favored the hypothesis that HSM2 and height have separate causal variants at this locus (PP = 98.3% and 1.7% for separate and shared signals, respectively). The 17q24.3 minor allele was associated with greater stature (*p* = 7 × 10^−6^), co‐localization analysis favoring distinct causal variants for HSM1 and height (PP = 76.5% and 23.2% for separate and shared signals respectively). The 4q31.21 minor allele was associated with smaller stature (*p* = 1.9 × 10^−6^), co‐localization analysis strongly favoring different causal variants for HSM2 and height (PP = 99.9%). The 12p11.22 minor allele was weakly associated with smaller stature (*p* = 0.01 for proxy SNP), co‐localization analysis strongly favoring different causal variants of HSM1 and height (PP = 97.8%). The minor allele of the *ASTN2* SNP was associated with greater stature (*p* = 1.4 × 10^−11^), co‐localization analysis favoring sharing of causal variants for HSM2 and height (PP = 93.9%).

**Table 2:** Single‐Nucleotide Polymorphism (SNP) Associations With Hip Osteoarthritis (OA), Femoral Neck (FN) Bone Mineral Density (BMD), Hip Fracture, and Anthropometric Traits

			Hip OA		Hip fracture		FN BMD		Height		Waist circumference	
Lead SNP	Gene/locus	EA	OR	*p*	OR	*p*	Beta	*p*	Beta	*p*	Beta	*p*
rs2158915	17q24.3	G	0.99^a^	0.64	**1.26**	**0.0029**	**–0.025**	**0.0017**	**–0.014**	**7 × 10^−6^**	–0.013	**0.0023**
rs1243579	14q32.13	G	1.01^a^	0.72	1.03^a^	0.71	**0.026**	**0.013**	–0.044^a^	0.25	–0.0057^a^	0.31
rs10743612	12p11.22	A	**1.14** ^a^	**9.6 × 10^−5^**	1.04	0.62	**–0.044**	**8.77 × 10^−7^**	**–0.0089**	**0.011**	–0.10	0.053
rs73197346	21q22.12	C	**0.87** ^b^	**0.006** ^b^	NA	NA	**–0.025**	**0.029**	NA	NA	NA	NA
rs59341143	4p15.33	C	1.00^a^	0.91	1.06^a^	0.54	NA	NA	–0.007^a^	0.1	–0.0041^a^	0.52
rs1966265	*FGFR4*	T	1.01	0.69	0.86	0.089	–0.016^a^	0.096	**0.035**	**3.8 × 10^−16^**	**0.029**	**3.9 × 10^−6^**
rs6537291	4q31.21	A	1.03^a^	0.26	0.93^a^	0.29	0.0092	0.24	**0.014** ^a^	**1.9 × 10^−6^**	–0.0024^a^	0.58
rs1885245	*ASTN2*	G	**1.09** ^a^	**0.0025**	**1.17**	**0.02**	0.00013	0.99	**0.02**	**1.4 × 10^−11^**	0.0029	0.5

Table shows other phenotypic associations for the genetic signals identified for hip shape illustrated in Table 1, including details of the effect (ie, minor) allele. Because SNPs identified in Table 1 were not genotyped/imputed consistently across other GWASs, a suitable proxy SNP (*r* ^2^ > 0.8) was selected by MRBase where necessary.^(a)^ Results show odds ratio and *p* value from the hip OA arcogen GWAS^16^ and UK Biobank GWAS,^(b)^ 20 and from an unpublished hip fracture GWAS. Beta estimates and *p* values are from GWAS for FN BMD,^19^ height,^17^ and waist circumference.^18^

^a^ Details of proxy SNPs used for each GWAS: Hip OA: rs2158915 proxied by rs8082221 (*r* ^2 ^= 1), rs1243579 proxied by rs3861665 (*r* ^2 ^= 0.878), rs10743612 proxied by rs258394 (*r* ^2^ = 0.865), rs59341143 proxied by rs10034452 (*r* ^2^ = 1), rs59341143 proxied by rs10034452 (*r* ^2 ^= 1), rs6537291 proxied by rs13118928 (*r* ^2 ^= 1), rs1885245 proxied by rs7856625 (*r* ^2 ^= 0.963); FN BMD: rs1966265 proxied by rs2011077 (*r* ^2 ^= 1); Height: rs1243579 proxied by rs3861665 (*r* ^2 ^= 0.878), rs59341143 proxied by rs10034452, rs6537291 proxied by rs2130339 (*r* ^2 ^= 1); Waist circumference: rs1243579 proxied by rs3861665 (*r* ^2 ^= 0.878), rs59341143 proxied by rs10034452, rs6537291 proxied by rs2130339; Fracture: rs1243579 proxied by rs1243577 (*r* ^2^ = 0.887), rs59341143 proxied by rs10034452 (*r* ^2^ = 1), rs6537291 proxied by rs7655625 (*r* ^2 ^= 0.97). Effect size reported for the minor allele for each proxy look‐up. NA means no suitable proxy could be found.

#### Hip OA

Minor alleles of proxy SNPs for the 12p11.22 and *ASTN2* SNPs were associated with greater risk of hip OA in arcOGEN^16^ (*p* = 9.6 × 10^−5^ and *p* = 0.0025, respectively) (Table 2). In addition, the 21q22.12 minor allele was associated with a reduced risk of hip OA in a recent GWAS based on UK Biobank (*p* = 0.006).^20^ Co‐localization analysis strongly favored a shared causal variant for HSM1 and hip OA at the 12p11.22 locus (PP = 99.6%) but not the 21q22.12 locus (PP = 32.8%). A shared causal variant between HSM2 and hip OA was also the favored hypothesis at the *ASTN2* locus (PP = 57.4%). In addition, rs13148031 and rs4837613, previously suggested to be associated with increased and decreased joint space width, respectively, are in high LD (*r* ^2^ > 0.8) with minor alleles of 4q31.21 and *ASTN2* reported here.^26^

#### Hip fracture

In an as yet unpublished GWAS (Supplemental Methods), the 17q24.3 and *ASTN2* minor alleles showed positive associations with hip fracture risk (*p* = 0.003 and *p* = 0.02, respectively). Co‐localization analysis favored a shared causal signal for hip shape and hip fracture at 17q24.3 (PP = 62.5% with HSM1) but not the *ASTN2* locus (PP = 23.6% with HSM2).

#### Other traits

The 17q24.3 minor allele was inversely related to femoral neck (FN) bone mineral density (BMD) (*p* = 0.002), co‐localization showing little evidence for a shared causal variant for HSM1 and FN BMD at this locus (PP = 13.7%). The 14q32.13, 12p11.22, and 21q22.12 SNPs were also associated with FN BMD (*p* = 0.013, *p* = 8.77 × 10^−7^ and *p* = 0.029, respectively). The 17q24.3 and *FGFR4* minor alleles were associated with smaller (*p* = 0.0023) and larger (*p* = 3.9 × 10^−6^) waist circumference, respectively.

### Sensitivity analyses

Genomewide significant signals were unchanged in HSM1 and HSM2 GWAS meta‐analyses including height adjustment (Supplemental Table S4).

### Functional evaluation

#### Gene function

Consistent with the above associations between loci that we identified and height, *FGFR4* is known to be involved in endochondral bone formation, as are SOX9, *PTHLH*, *HHIP*, *NKX3‐2*, *DICER1*, and *RUNX1* within 200 kbp of 17q24.3, 12p11.22, 4q31.21. 4p15.33, 14q32.13, and 21q22.12, respectively (Table 3). These genes are also associated with Mendelian disorders causing skeletal abnormalities and/or skeletal defects in murine knockouts (Table 3).

**Table 3:** Function of Genes Linked to the Single‐Nucleotide Polymorphisms (SNPs) Associated With Hip Shape

Locus	Osteoblast/chondrocyte function	Mendelian disorder causing skeletal abnormalities	Skeletal defect in murine knockout
*SOX9* (17q24.3, rs2158915: upstream)	Transcription factor primarily involved in chondrogenesis,^42^ SOX9 activity can be increased through being targeted by PTHLH^33^	Campomelic dysplasia,^43^Pierre Robin sequence,^44^Cooks syndrome^45^	Chondrodysplasia and abnormal joint formation^46^
GSC (14q32.13, rs1243579: upstream)	Goosecoid Homeobox, Transcription factor involved in early development^27^	Short stature, auditory canal atresia, mandibular hypoplasia, and skeletal abnormalities^27^	Severe craniofacial abnormalities^47^
*DICER1* (14q32.13, rs1243579: downstream)	Ribonucleases III, which cleaves mRNA into microRNA, involved in regulating chondrocyte proliferation^37^	None identified	Inhibition of chondrocyte proliferation resulting in skeletal growth abnormalities^37^
*PTHLH* (12p11.22, rs10743612: downstream)	Parathyroid hormone‐like hormone, regulates endochondral bone development,^34^inhibits chondrocyte differentiation^34^	Brachydactyly^48^	Dyschondroplasia^49^
*RUNX1* (21q22.12, rs73197346: upstream)	Interacts with RUNX2 to regulate endochondral bone formation^38^	None identified	Abnormal chondrogenesis of the sternum and skull^50^
*NKX3‐2* (4p15.33, rs59341143: downstream)	NK3 Homeobox 2, promotes chondrogenesis by inhibiting factors that interact with bone morphogenetic proteins (BMPs)^36^	Spondylo‐megaepiphyseal‐metaphyseal dysplasia^51^	Vertebral defects^52^
*FGFR4*	FGR4 regulates chondrocyte autophagy^32^	None identified	Impaired long bone
*HHIP* (4q31.21, rs6537291: downstream)	Ihh regulates chondrocyte differentiation and is critical in endochondral bone formation^35^	Brachydactyly^53^	Shortened skeleton (overexpression in chondrocytes)^54^
*ASTN2*	None identified	None identified	No mouse knockouts described

Table summarizes the role of genes related to SNPs identified in Table 1. rs1966265 and rs1885245 reside within the gene of interest; the other SNPs are intergenic. For the latter, the nearest protein coding gene(s) was selected as gene(s) of interest, and role in osteoblast and/or chondrocyte function and skeletal expression summarized, along with whether the gene is known to cause a Mendelian disease (curated from OMIM) associated with a skeletal abnormality, or a skeletal phenotype when knocked out in mice.

#### Osteoblast eQTL look‐up

The 14q32.13 SNP showed evidence of cis‐regulatory activity in a human osteoblast eQTL database (*GSC* [*p* = 0.0012], *SERPINA10* [*p* = 0.0042], *ASB2* [*p* = 0.017], *TCL6* [*p* = 0.041]), of which mutations in *GSC* (Goosecoid Homeobox) have previously been reported to cause hip dysplasia.^27^ *p*_HEIDI_ > 0.05 confirmed that a single causal variant within the 14q32.13 LD block is likely to affect both osteoblast GSC expression and HSM1. The 12p11.22 SNP also showed evidence of cis‐regulatory activity in human osteoblasts, though none of the associated genes are of known functional relevance (*REP15* [*p* = 0.0016], *FGFR1OP2* [*p* = 0.012], *PPFIBP1* [*p* = 0.016]).

RegulomeDB was used to screen SNPs in LD with lead SNPs for predicted functional consequences. At 14q32.13, rs12436596, in perfect LD with rs1243579 and with equivalent eQTL associations, showed strong evidence of functionality in RegulomeDB and also Haploreg and CADD, as well as evolutionary conservation in GERP++ (Supplemental Table S5). SNPs in LD with the majority of other lead SNPs were also identified with predicted functional consequences by RegulomeDB and haploreg.

ATAC‐seq was employed to examine intersections between SNPs in LD with lead variants and sites of open chromatin, depicting putative regulatory sequences, in DNA derived from mouse embryonic proximal femora. Intersections were observed for three SNPs: rs28718249 on chromosome 4, *r* ^2^ = 0.51 with the lead variant (rs59341143), at an ATAC‐seq peak immediately adjacent to the *RAB28* promoter; rs6871994 on chromosome 5, *r* ^2^ = 0.98 with the lead variant (rs17725170), at an ATAC‐seq peak within a regulatory gene desert; rs4836757, located within an *ASTN2* intron 9, *r* ^2 ^= 0.52 with the lead variant (rs1885245) (Supplemental Fig. S12).

To look for enrichment of open chromatin signals within lead variant locus, a randomized set of matched loci was generated and intersected with the ATAC‐seq data from proximal femur. This indicated a significant (*p* < 0.05) overlap of our lead variant locus with putative regulatory regions, an enrichment not observed with an ATAC‐seq data set from brain, nor active chromatin mark (H3K27ac) data from human bone marrow–derived cells and developing limb buds. Additionally, enrichment testing was performed for lead variants associated with individual hip‐shape measures—although no sets showed enrichment using a Bonferroni‐corrected genomewide significance threshold of 10^−9^, HSM1 had a significant overlap with proximal femur ATAC‐seq peaks (*p* < 0.05) when using a lower‐stringency threshold of 10^−7^.

## Discussion

This GWAS of hip shape identified nine SNPs, in eight loci, to be associated with hip shape by GWAS meta‐analysis at Bonferroni‐corrected genomewide significance (*p* < 5 × 10^−9^). Consistent signals were observed across the five cohorts despite their differences in age, sex, and type of DXA scanner used. Genetic associations were identified for three of the 10 modes examined (ie, HSM1, HSM2, and HSM5). It may be that the remaining HSMs, which together explain approximately 50% of total variance in hip shape, are less heritable, consistent with our finding that, apart from HSM1, HSM2, and HSM5, only HSM8 showed an SNPwise heritable component by LD score regression.

Although this represents the first reported GWAS for hip shape, previous candidate gene studies^7^, ^8^ have examined associations between OA susceptibility loci and hip shape, following application of SSM to radiographs (Supplemental Table S6). rs4836732, reported to be associated with hip shape by Lindner and colleagues,^6^ is in LD (*r* ^2 ^= 0.49) with rs1885245 *ASTN2* SNP we found to be associated with HSM2, both studies observing relationships with femoral head size. Six other SNPs identified across these two SSM studies showed little evidence of association with hip shape in the present study. In a more recent candidate gene study, we observed a similar relationship between rs10492367 and HSM1 in ALSPAC mothers to that found here for rs10743612/12p11.22, with which it is in high LD (*r* ^2 ^= 0.78; Supplemental Fig. S6).^26^ Therefore, using the hypothesis‐free GWAS approach, the present study has advanced understanding of the genetic influence on hip shape through identification of six new associated loci.

The shape effects may have pathological sequelae, as suggested by evidence that associations between the 12p11.22 and *ASTN2* locus SNPs, and HSM1 and HSM2, respectively, co‐localized with signals previously reported in association with hip OA risk.^16^ This implied relationship between hip shape and hip OA is in‐keeping with reported associations between hip DXA‐derived hip shape, obtained using the same SSM method as used here, and hip OA.^8^, ^9^ For example, a wider upper femur as reflected by HSM1, associated with 12p11.22 and 21q22.12 loci related to hip OA, might alter biomechanical forces of the hip and hence risk of hip OA. Femoral head size and shape, related to HSM2 (Supplemental Fig. S1), might likewise affect risk of hip OA by influencing hip biomechanics. However, individual HSMs are associated with variation in multiple aspects of hip shape, and precisely which aspect is responsible for pathological sequelae such as hip OA is currently unknown.

The genetic influences on hip shape that we identified may also influence fracture risk, given evidence of co‐localization of genetic signals for HSM1 and hip fracture for the 17q24.3 locus. 17q24.3, which is upstream of *SOX9*, is also associated with known risk factor for hip fracture, FN BMD.^28^ However, this appears to represent a distinct genetic signal to that associated with HSM1. Several other geometric parameters independent of BMD have previously been found to predict risk of hip fracture, including hip axis length, neck shaft angle, and cross‐sectional moment of inertia.^29^, ^30^ However, SNPs at the 17q24.3 locus showed little relationship with geometric parameters in a hip structural analysis (HSA) GWAS performed on an overlapping set of DXA images,^31^ suggesting that genetic influence on hip fracture acting via hip shape are not solely explained by relationships with known geometric variables.

Because BMD, shape, and geometry are all derived from hip DXA scans, some shared genetic dependency is expected. Consistent with this, four hip shape‐associated loci were also associated with femoral neck BMD, based on a look‐up of a previously published GWAS. In addition, there was some overlap with the above HSA GWAS after height adjustment, particularly for HSM2‐associated loci. For example, rs17725170 was in high LD (*r* ^2^ > 0.8) with a SNP associated with femoral neck length at genomewide significance, and rs1966265 in weak LD (*r* ^2^ > 0.3) with a SNP associated with narrow neck width at genomewide significance (David Karasik and colleagues, unpublished observations). Further analyses are warranted to explore shared genetic heritability between these distinct hip DXA‐derived traits.

Further work is necessary to determine or confirm the exact genetic variants implicated by these GWAS results. Some candidates look promising; for example, the HSM1 GWAS signal at 14q32.13 associated with osteoblast expression of GSC, a transcription factor involved in early skeletal development. Loss‐of‐function mutations in this same gene have previously been shown to be associated with bilateral femoral head dysplasia.^27^ SNPs at 17q24.3, 12p11.22, *FGFR4*, 4q31.21, and *ASTN2* were associated with height as well as hip shape, co‐localization analysis demonstrating sharing of the same causal signal for hip shape and height in the case of the *ASTN2* locus. In terms of the basis for these associations between identified hip shape loci and height, rs1966265 is a non‐synonymous variant in *FGFR4*, which is involved in endochondral bone formation.^32^ Furthermore, the 17q24.3, 12p11.22, 4q31.21, 4p15.33, 14q32.13, and 21q22.12 loci are all in close proximity to known regulators of endochondral bone formation, namely *SOX9*,^33^ *PTHLH*,^34^ *HHIP*,^35^ NKX3‐2,^36^ *DICER1*,^37^ and *RUNX1*,^38^ respectively. A link between limb growth and hip shape is consistent with previous findings that canine hip dysplasia is more common in larger breeds that grow more rapidly.^39^ Although there is currently little evidence to support a similar relationship between height and hip development in humans, this might explain the relationship between height and hip OA risk reported in epidemiological studies.^40^, ^41^

Several proxy SNPs for the lead variants were identified with potential impacts on function. For example, we identified a proxy SNP at 14q32.13, which was strongly related to GSC expression in osteoblasts, predicted by haploloreg to act as an enhancer, and showed evidence of evolutionary conservation. ATAC‐seq also suggested an overall enrichment of our lead variant loci for interections with putative regulatory regions.

### Strengths and weaknesses

This study represents the first hip shape GWAS, leading to the identification of several novel loci for this phenotype, which may in turn reveal novel pathogenic pathways for hip fracture and OA. Because our GWAS was limited to the first 10 HSMs, which explain approximately 85% of total variance in hip shape, our analyses effectively excluded 15% of variance in hip shape. In terms of other weaknesses, as in many meta‐analyses, there were some inconsistencies in data collection between cohorts. For example, the aspect ratio of scans from the Lunar Prodigy used in ALSPAC differed from that of other scanners; however, because GWAS was performed at the level of individual cohorts, this limitation is unlikely to have affected our results as the heterogeneity was low (*I* ^2^ ranged from 0 to 0.15). A further limitation is the reliance on hip DXA scans for GWAS studies of hip shape in large population‐based cohorts. Whereas newer generations of DXA scanners have relatively high levels of resolution, this was not the case for older devices used to acquire hip DXA scans from participating cohorts, in which resolution is relatively poor, leading to difficulty in accurately defining characteristics such as osteophytes. Nevertheless, we replicated at least one genetic association with hip shape previously identified from radiographic studies. In addition, given the sample size, we elected to focus on common SNPs (MAF ≥ 1%), which may have overlooked important genetic influence from lower‐frequency SNPs exerting relatively strong effects.^19^

Lack of a separate replication cohort represents a further limitation to the present study, particularly in the case of those associations that were close to the *p* < 5 × 10^−9^ genomewide significance. Because PCA methods that we employed generate shape phenotypes specific to the data set, this potentially limits the opportunity for independent replication in other cohorts. That said, HSM1, reflecting upper femur height‐to‐width ratio, for which we observed the largest number of genetic associations, is consistently the first mode generated by SSM in DXA‐based hip images, irrespective of which study population this is applied to. Moreover, parameters for the shape model used in this study are available on request, making it feasible to generate identical shape parameters in other cohorts, enabling independent replication of our findings.

## Conclusions

A GWAS meta‐analysis identified eight loci consistently associated with DXA‐derived hip shape. Most loci were also associated with height and close to genes involved in endochondral bone formation, pointing to a relationship between genetic influence on hip shape and limb growth. An additional locus associated with HSM1, immediately upstream of the *GSC* gene implicated in hip dysplasia, had evidence of cis‐regulatory activity in osteoblasts. Further studies are justified to identify additional hip shape loci, by analyzing rare variants and regional hip models, and to determine the mechanisms by which genetic variation in hip shape produces pathological consequences such as hip OA and hip fracture.

## Disclosures

All authors state that they have no conflicts of interest.

## Supporting information

Supporting Information S1.

Supporting Data S1.
