# Supplementary material for: Identification of Novel Loci Associated With Hip Shape: A Meta‐Analysis of Genomewide Association Studies
Source: J Bone Miner Res. 2018 Nov 26;34(2):241–51. doi: 10.1002/jbmr.3605 (PMC6375741; doi:10.1002/jbmr.3605)
Supplement: Supplementary file 1 — Supporting Information S1. [file JBMR-34-241-s001.docx]

**SUPPLEMENTARY METHODS**

**Participant details, DXA and Genotyping methods for each hip shape cohort**

ALSPAC is a longitudinal birth cohort that recruited pregnant women resident in a geographical area in the South West of England, UK, with an expected delivery date between April 1, 1991, and December 31, 1992. The present study uses data from follow-up research clinics of mothers between 2008 and 2011 (1, 2). All eligible mothers (*i.e.* still engaged with the study; alive with known contact details and who had not withdrawn their consent) were invited to these assessments. Of 11,264 (82%) women invited, 4,834 (43%) attended. Informed consent was collected. Ethical approval for the study was obtained from the ALSPAC Ethics and Law Committee and the Local Research Ethics Committees (North Somerset & South Bristol Research Ethics Committee: 08/H0106/96). Please note that the study website contains details of all the data that is available through a fully searchable data dictionary (http://www.bris.ac.uk/alspac/researchers/data-access/data-dictionary/). Hip dual-energy X-ray absorptiometry (DXA) scans, performed by GE Lunar Prodigy Scanner (Madison, WI, USA), were acquired and analysed according to manufacturer’s standard scanning and positioning protocol. SNPs with a genotype missingness rate greater than 5%, or extreme deviation from Hardy-Weinberg equilibrium (P < 5x10^-7^) were removed. Individuals with extreme heterozygosity, evidence of population stratification and cryptic relatedness (IBD > 0.125) were excluded. Details of this pipeline are reported elsewhere (3). Genotypic data were phased using ShapeIT version 2 and imputed on IMPUTE version 2.2.2 using the Haplotype Reference Consortium (HRC) version 1 panel.

MrOS is a prospective study of 5,994 men recruited between 2000-2002 at six centers around the United States (Birmingham, Alabama; Minneapolis, Minnesota; Palo Alto, California; the Monongahela Valley near Pittsburgh, Pennsylvania; Portland, Oregon; and San Diego, California). To be eligible, men had to be ≥ 65 years old, ambulatory, and without bilateral hip replacements. A full description of the MrOS cohort has been previously published (4). We used hip shape data derived from DXA scans performed at the baseline visit. Right hip DXA imaging was performed at the baseline visit unless they had a right hip replacement in which case a left hip scan was performed. A QDR 4500 Hologic machine (Waltham, MA) was used at all six sites.

Genomic DNA from participants in the MrOS Study was extracted from whole blood samples collected at the baseline visit as previously reported(5). 739,528 SNPs passed QC in 4615 samples of European ancestry that were used for genotype imputation. Prior to imputation, scripts developed by Will Rayner (<http://www.well.ox.ac.uk/~wrayner/tools>) were used to compare MrOS genotype data with genotype data from 1000 Genomes Phase 3 EUR samples. Strand on which alleles were represented were updated to match the reference panel. SNPs were removed if they were coded with A/T or G/C alleles with MAF>0.4, SNP alleles differed from the reference panel, SNPs were absent from reference panel, or if the allele frequency differed by more than 0.2 from the EUR samples in the reference panel. Genotype imputation was performed using the University of Michigan imputation server(6). Sample genotypes were phased using ShapeIt2, and imputation was performed using minimac3. Reference panel used was 1000 Genomes Phase 1 version 3. Genotype dosage was extracted from VCF files using DosageConvertor to produce PLINK dosage files (https://genome.sph.umich.edu/wiki/DosageConvertor). Genetic association analysis was performed using PLINK v1.9.

SOF The Study of Osteoporotic Fractures (SOF) is a prospective multicenter study of risk factors for vertebral and non vertebral fractures (7). The cohort is comprised of 9704 community dwelling women 65 years old or older recruited from populations-based listings in four U.S. areas: Baltimore, Maryland; Minneapolis, Minnesota; Portland, Oregon; and the Monongahela Valley, Pennsylvania. Women enrolled in the study were 99% Caucasian with African American women initially excluded from the study due to their low incidence of hip fractures. A cohort of AA women was recruited at the 6^th^ Visit. The inclusion criteria were: 1) 65 years or older, (2) ability to walk without the assistance of another, (3) absence of bilateral hip replacements, (4) ability to provide self-reported data, (5) residence near a clinical site for the duration of the study, (6) absence of a medical condition that (in the judgment of the investigator) would result in imminent death, and (7) ability to understand and sign an informed consent. To qualify as an enrollee, the participant had to provide written informed consent, complete the self-administered questionnaire (SAQ), attend the clinic visit, and complete at least the anthropometric measures. The SOF study recruited only women. Dual X-ray absorptiometry (DXA) was performed on participants at Visit 2 using Hologic QDR 1000 workstations at each of the four clinics. Hip and spine scans were performed.

In collaboration with Roche Molecular Systems (Alameda, CA), DNA from participants of the Study of Osteoporotic Fractures (SOF) was extracted from either buffy coat or whole blood samples collected at either visit 2 (1989–1990) or visit 6 (1997–1998). As previously reported, among the 9704 SOF participants enrolled at the baseline visit, following QC, 3924 samples had sufficient DNA quantity and DNA solution volume and underwent whole-genome genotyping(5). All DNA samples eligible for whole-genome genotyping were genotyped using Sequenom iPLEX technology for a 24-SNP “fingerprint” panel. Genotyping and QC as performed for MrOS were also applied to SOF samples with the following exceptions: heterozygous genotypes on the X were not set to missing, In SOF, 739,528 SNPs in 3625 samples of European ancestry that passed QC were used for genotype imputation. Pre-imputation QC using scripts from Will Rayner, genotype imputation, post-imputation data formatting, and association analysis was performed in the same manner as for the MrOS samples.

FOS Details and descriptions of the Framingham Osteoporosis Study (FOS) have been reported previously (8) and are available publicly through the Database of Genotype and Phenotype (dbGaP) at <http://view.ncbi.nlm.nih.gov/dbgap>. In brief, the Original and the Offspring cohorts in the FOS represent members of two-generational (mostly nuclear) families recruited at different times. The study was approved by the Institutional Review Boards for Human Subjects Research of Boston University and Hebrew SeniorLife. The participants underwent bone densitometry by dual-energy X-ray absorptiometry (DXA) with a Lunar DPX-L device (Lunar Corp., Madison, WI, USA) between 1996 and 2001. All genome-wide genotyping has been previously reported(9). Genotypes were called using the Illumina’s BeadStudio calling algorithm. The sample quality control exclusion criteria were sample call rate < 97%, excessive autosomal heterozygosity, first and second degree relatives, genotypic sex mismatch using X and Y chromosome probe intensities and gross chromosome abnormalities. The genotypes were imputed using the HRC reference panel and pipeline.

TwinsUK The TwinsUK cohort is comprised of ~12,000 monozygotic and dizygotic twins recruited without selection for any particular trait from St Thomas’ UK Adult Twin Registry (TwinsUK) (www.twinsuk.ac.uk/). This cohort is from Northern European/UK ancestry and has been shown to be representative of singleton populations and the UK population in general(10). Medical history and lifestyle-factor data were obtained using detailed health questionnaires, with exclusion criteria applied including rheumatoid arthritis, oral steroid use or surgical oophorectomy. All study participants provided written, informed consent and the research was approved by the Guy’s and St Thomas' Hospital Research Ethics Committee. Hip DXA scans for the TwinsUK cohort were performed using a Hologic QDR 4500W (Bedford, MA, USA) using standard manufacturer protocols. DNA for genotyping was extracted from whole blood samples obtained for the vast majority of the cohort at the time of the study visit. Genotyping was completed for 5,654 individuals using the Illumina HumanHap300, HumanHap610, 1M-Duo and 1.2M-Duo arrays, as described previously (11). Genotype imputation was performed using the HRC release 1.1 reference panel in conjunction with the Minimac3 software (Michigan Imputation Server).

**UFO Hip Fracture GWAS**

The Umeå Fracture and Osteoporosis (UFO) study is a population-based study designed to identify the genetic and environmental determinants of osteoporotic fractures. This cohort is sampled from a longitudinal, population-based cohort study from Northern Sweden (the Northern Sweden Health and Disease Study, NSHDS), consisting of blood samples, lifestyle- and dietary data from ~ 100,000 unique subjects from the county of Västerbotten (~ 255,000 inhabitants as of Dec 31^st^ 2007).(12, 13) Hip fracture cases were identified by merging the NSDHS-cohort with medical records and radiographic reports. During a 15 years period 1993-2008, we identified 1086 subjects with hip fractures that were also represented with a DNA sample in the biobank. The inclusion criteria for the cases in the UFO-hip GWAS fracture cohort were subjects > 20 years of age, suffering from a hip fracture defined by medical records and/or radiograph reports. The UFO-hip GWAS fracture controls were drawn from the controls of a previous GWAS study of glioma(14) and were only excluded in our hip GWAS fracture study if they had suffered a previous hip and/or forearm fracture. The fracture cases were compared with a set of 934 controls, making a total of 2020 subjects (1941 of these passed quality check for genotyping). The UFO study was approved by the local research ethics committee at Umeå University. Written consent was obtained from all participants.

Genotyping of both the hip fracture cases and controls was performed with the Illumina HumanHap660 arrays (Cases at Department of Internal Medicine, Erasmus Medical Center, Rotterdam, the Netherlands and the controls at the Core Genotyping facility at National Cancer Institute, Bethesda, USA). The quality controls and imputations of both cases and controls were performed simultaneously at the Genetic Laboratory, Department of Internal Medicine, Erasmus Medical Center, Rotterdam, the Netherlands. Genotypes were called using the BeadStudio calling algorithm. Genotypes from 1941 individuals passed the sample quality control criteria [exclusion criteria: sample call rate <97.5%, gender discrepancy with genetic data from X-linked markers, excess autosomal heterozygosity >0.33 (~FDR<0.1%), duplicates and/or first degree relatives identified using IBS probabilities (>97%), ethnic outliers (3 SD away from the population mean) using multi-dimensional scaling analysis with four principal components]. We carried out imputation to HapMap release 22 (after excluding SNPs with MAF<1%, SNP call rate <98% and HWE p value <1×10^−6^) using Minimac and Mach 1.0, *Markov Chain Haplotyping*, giving a total of 2,436,060 SNPs.

**Functional SNP annotation**

RegulomeDB was used to identify additional SNP(s) in LD which were likely to have functional consequences, using an LD r^2^ cut-off of 0.6 (none of the lead SNPs were predicted to have functional consequences in RegulomeDB) (15). Further functional annotation was performed using a combination of HaploReg for exploring regulatory motifs (16), GERP ++ to identify regions under evolutionary constraint (17), and Combined Annotation Dependent Depletion (CADD) for integrating a range of annotations into a single measure (18). Functional consequences of exonic SNPs identified from GWAS were evaluated with PolyPhen2 (19).

**ATAC-seq**

Lead SNP sets were analyzed with Proxyfinder to identify proxy SNPs from the European subset of 1000 Genomes Phase 3 (20), using an LD r^2^ cutoff of 0.5, a minimum MAF of 0.01, and a window size of 500kb, with other default settings. For the set of 12 lead variants, this analysis yielded 694 proxy SNPs. Next, a published dataset, generated through the Assay for Transposase-Accessible Chromatin followed by sequencing (ATAC-seq) (21), from mouse proximal femur (consisting of the head, neck, trochanter, and proximal growth plate, up to but excluding the osseous diaphysis) was used to demarcate open chromatin regions in the developing femur(22). This dataset was processed as previously described, resulting in a final set of 24,804 called peaks which were replicate consolidated (N = 2 biological replicates) using an IDR (irreproducible discovery rate) cutoff of 0.05. Raw sequencing data as well as processed peak.bed files were previously deposited under NCBI GEO accession GSE100585. For intersection with human hip-shape loci this peak set was transferred from mm10 to hg19 using the liftOver utility from UCSC using the ‘minMatch=0.1’ option. All intersections were done using BEDTools v2.25.0 (23) using a minimum overlap of 1bp.

SNPSNAP (24) was next used to generate 1000 random-matched sets of loci based on the original lead SNP set. The European subset of 1000 Genomes Phase 3 (20) was used for SNPSNAP, along with an r^2^ cutoff of 0.5, with other default settings. Similar to the lead set, proxy SNPs for random-matched loci sets were generated with ProxyFinder. For the enrichment analysis peaks were intersected with proxy SNPs using BEDTools v2.25.0, with overlaps counted per-locus and summed over a SNP set to obtain intersection numbers for testing. Peak set intersections were done for proxy SNPs of a lead SNP set, as well as proxies for the 1000 random-matched sets of loci to establish a background - counts were standardized and significance of enrichment was assessed using a continuous distribution function of the standard normal distribution implemented in R3.4.2 (25). Additional independent datasets were analyzed: H3K27ac ChIP-seq data from human E41 limb bud was obtained from Cotney et al. 2013 (26), along with H3K27ac data from ENCODE BMDCs (27, 28). For these datasets, peak calling was done with MACS2 (29) with replicate data consolidated using an IDR cutoff of 0.05. Enrichment testing for additional epigenetic datasets was performed as above (results available upon request). Finally, proxy SNPs were also obtained for lead variants identified at a less stringent genome-wide significance threshold (10^-7^) associated with individual hip-shape measures. Random-matched SNP sets were generated as above using SNPSNAP for lead variants associated with each hip-shape measure individually, and when aggregating all lead variants together. Significance testing for ATAC-seq overlap enrichment was done as above for all sets.

**References**

1. Boyd A, Golding J, Macleod J, Lawlor DA, Fraser A, Henderson J, et al. Cohort Profile: the 'children of the 90s'--the index offspring of the Avon Longitudinal Study of Parents and Children. Int J Epidemiol. 2013;42(1):111-27.

2. Fraser A, Macdonald-Wallis C, Tilling K, Boyd A, Golding J, Davey Smith G, et al. Cohort Profile: The Avon Longitudinal Study of Parents and Children: ALSPAC mothers cohort. Int J Epidemiol. 2013;42(1):97-110.

3. Evans DM, Zhu G, Dy V, Heath AC, Madden PA, Kemp JP, et al. Genome-wide association study identifies loci affecting blood copper, selenium and zinc. Hum Mol Genet. 2013;22(19):3998-4006.

4. Blank JB, Cawthon PM, Carrion-Petersen ML, Harper L, Johnson JP, Mitson E, et al. Overview of recruitment for the osteoporotic fractures in men study (MrOS). Contemp Clin Trials. 2005;26(5):557-68.

5. Evans DS, Cailotto F, Parimi N, Valdes AM, Castano-Betancourt MC, Liu Y, et al. Genome-wide association and functional studies identify a role for IGFBP3 in hip osteoarthritis. Ann Rheum Dis. 2015;74(10):1861-7.

6. Das S, Forer L, Schonherr S, Sidore C, Locke AE, Kwong A, et al. Next-generation genotype imputation service and methods. Nat Genet. 2016;48(10):1284-7.

7. Cummings SR, Nevitt MC, Browner WS, Stone K, Fox KM, Ensrud KE, et al. Risk factors for hip fracture in white women. Study of Osteoporotic Fractures Research Group. N Engl J Med. 1995;332(12):767-73.

8. Hannan MT, Felson DT, Dawson-Hughes B, Tucker KL, Cupples LA, Wilson PW, et al. Risk factors for longitudinal bone loss in elderly men and women: the Framingham Osteoporosis Study. J Bone Miner Res. 2000;15(4):710-20.

9. Zheng HF, Forgetta V, Hsu YH, Estrada K, Rosello-Diez A, Leo PJ, et al. Whole-genome sequencing identifies EN1 as a determinant of bone density and fracture. Nature. 2015;526(7571):112-7.

10. Spector TD, Williams FM. The UK Adult Twin Registry (TwinsUK). Twin Res Hum Genet. 2006;9(6):899-906.

11. Metrustry SJ, Edwards MH, Medland SE, Holloway JW, Montgomery GW, Martin NG, et al. Variants close to NTRK2 gene are associated with birth weight in female twins. Twin Res Hum Genet. 2014;17(4):254-61.

12. Englund U, Nordstrom P, Nilsson J, Bucht G, Bjornstig U, Hallmans G, et al. Physical activity in middle-aged women and hip fracture risk: the UFO study. Osteoporosis international : a journal established as result of cooperation between the European Foundation for Osteoporosis and the National Osteoporosis Foundation of the USA. 2011;22(2):499-505.

13. Hallmans G, Agren A, Johansson G, Johansson A, Stegmayr B, Jansson JH, et al. Cardiovascular disease and diabetes in the Northern Sweden Health and Disease Study Cohort - evaluation of risk factors and their interactions. Scand J Public Health Suppl. 2003;61:18-24.

14. Rajaraman P, Melin BS, Wang Z, McKean-Cowdin R, Michaud DS, Wang SS, et al. Genome-wide association study of glioma and meta-analysis. Human genetics. 2012;131(12):1877-88.

15. Boyle AP, Hong EL, Hariharan M, Cheng Y, Schaub MA, Kasowski M, et al. Annotation of functional variation in personal genomes using RegulomeDB. Genome Res. 2012;22(9):1790-7.

16. Ward LD, Kellis M. HaploReg: a resource for exploring chromatin states, conservation, and regulatory motif alterations within sets of genetically linked variants. Nucleic Acids Res. 2012;40(Database issue):D930-4.

17. Davydov EV, Goode DL, Sirota M, Cooper GM, Sidow A, Batzoglou S. Identifying a high fraction of the human genome to be under selective constraint using GERP++. PLoS Comput Biol. 2010;6(12):e1001025.

18. Kircher M, Witten DM, Jain P, O'Roak BJ, Cooper GM, Shendure J. A general framework for estimating the relative pathogenicity of human genetic variants. Nat Genet. 2014;46(3):310-5.

19. Adzhubei I, Jordan DM, Sunyaev SR. Predicting functional effect of human missense mutations using PolyPhen-2. Curr Protoc Hum Genet. 2013;Chapter 7:Unit7 20.

20. Auton A, Brooks LD, Durbin RM, Garrison EP, Kang HM, Korbel JO, et al. A global reference for human genetic variation. Nature. 2015;526(7571):68-74.

21. Buenrostro JD, Giresi PG, Zaba LC, Chang HY, Greenleaf WJ. Transposition of native chromatin for fast and sensitive epigenomic profiling of open chromatin, DNA-binding proteins and nucleosome position. Nat Methods. 2013;10(12):1213-8.

22. Guo M, Liu Z, Willen J, Shaw CP, Richard D, Jagoda E, et al. Epigenetic profiling of growth plate chondrocytes sheds insight into regulatory genetic variation influencing height. Elife. 2017;6.

23. Quinlan AR, Hall IM. BEDTools: a flexible suite of utilities for comparing genomic features. Bioinformatics. 2010;26(6):841-2.

24. Pers TH, Timshel P, Hirschhorn JN. SNPsnap: a Web-based tool for identification and annotation of matched SNPs. Bioinformatics. 2015;31(3):418-20.

25. R Core Team. R: A language and environment for statistical computing. R Foundation for Statistical Computing, Vienna, Austria. 2017 [Available from: <https://www.R-project.org/>.

26. Cotney J, Leng J, Yin J, Reilly SK, DeMare LE, Emera D, et al. The evolution of lineage-specific regulatory activities in the human embryonic limb. Cell. 2013;154(1):185-96.

27. Encode Project Consortium An integrated encyclopedia of DNA elements in the human genome. Nature. 2012;489(7414):57-74.

28. Kundaje A, Meuleman W, Ernst J, Bilenky M, Yen A, Heravi-Moussavi A, et al. Integrative analysis of 111 reference human epigenomes. Nature. 2015;518(7539):317-30.

29. Zhang Y, Liu T, Meyer CA, Eeckhoute J, Johnson DS, Bernstein BE, et al. Model-based analysis of ChIP-Seq (MACS). Genome Biol. 2008;9(9):R137.
