# Supplementary material for: Identification of Novel Loci Associated With Hip Shape: A Meta‐Analysis of Genomewide Association Studies
Source: J Bone Miner Res. 2018 Nov 26;34(2):241–51. doi: 10.1002/jbmr.3605 (PMC6375741; doi:10.1002/jbmr.3605)

**SUPPLEMENTARY RESULTS**

**FIGURE LEGENDS**

Supplementary Figure 1: Plots showing shape changes for the first 10 HSMs, generated in SHAPE. Dotted line: -2SD; solid line: +2SD.

Supplementary Figure 2: Meta-analysis QQ plots for each of the first 10 hip shape modes. Genomic Inflation Factors: λ_1_= 1.07, λ_2_ =1.06, λ_3_ = 1.02_,_ λ_4_ = 1.02_,_ λ_5_ = 1.06, λ_6_ = 1.03, λ_7_ = 1.03, λ_8_  = 1.05, λ_9_ = 1.03, λ_10_ **=** 1.02.

Supplementary Figure 3: Manhattan plots for the modes showing genome-wide significant genetic associations (HSM1, HSM2 and HSM5).

Supplementary Figure 4: Regional association plot for rs2158915 locus associations with (A) HSM1 and (B) HSM5.

Supplementary Figure 5: Regional association plot for rs1243579 locus association with HSM1.

Supplementary Figure 6: Regional association plot for rs10743612 locus association with HSM1.

Supplementary Figure 7: Regional association plot for rs73197346 locus association with HSM1.

Supplementary Figure 8: Regional association plot for rs59341143 locus association with HSM1.

Supplementary Figure 9: Regional association plot for rs1966265 locus association with HSM2

Supplementary Figure 10: Regional association plot for rs1542725 locus association with HSM2.

Supplementary Figure 11: Regional association plot for rs1885245 locus association with HSM2.

Supplementary Figure 12: Lead variant loci intersecting regions of open chromatin during proximal femur development. All panels indicate intersections between proxy SNPs in LD (r^2^ > 0.5; 1000G EUR) to lead GWAS variants (“Hip-shape Proxy SNPs”) and ATAC-seq data generated from E15.5 proximal femur tissue (“Proximal Femur ATAC-seq”), visualized using the UCSC Genome Browser (1) in hg19. Gene annotations within and adjacent to regions of interest are indicated (“UCSC Genes”). Below the subtitle “100 vertebrates Basewise Conservation by PhyloP”, a track is shown indicating a measurement of per-bp conservation across 100 vertebrate species based on phyloP (2) (See UCSC genome browser for conservation display details). In each panel, the vertical red line indicates the proxy SNP intersecting an ATAC-seq peak. (A) Proxy SNP for rs59341143 and Proximal Femur ATAC-seq peak intersection upstream of *RAB28* promoter. (B) Proxy SNP for rs17725170 and Proximal Femur ATAC-seq peak intersection within a gene desert on chromosome 5. (C) Proxy SNP for rs1885245 and Proximal Femur ATAC-seq peak intersection within an intronic region of *ASTN2*. (1) Kent WJ, Sugnet CW, Furey TS, Roskin KM, Pringle TH, Zahler AM, Haussler D. (2002) [The human genome browser at UCSC](http://www.genome.org/cgi/content/abstract/12/6/996). *Genome Res.* 12(6):996-1006. (2) Pollard, K. S., Hubisz, M. J., Rosenbloom, K. R., & Siepel, A. (2010). Detection of nonneutral substitution rates on mammalian phylogenies. *Genome Res.*, 20(1), 110-121.

**Supplementary Table 1: HSMs in participating cohorts. HSMs are expressed as deviation from the mean shape in the combined hip shape sample in standard normal units (mean=0, SD=1)**

|  | **ALSPAC** | | **FHS** | | **MrOS** | | **SOF** | | **Twins** | |
| --- | --- | --- | --- | --- | --- | --- | --- | --- | --- | --- |
|  | **Mean** | **SD** | **Mean** | **SD** | **Mean** | **SD** | **Mean** | **SD** | **Mean** | **SD** |
| HSM1 | 1.44 | 0.54 | 0.22 | 0.45 | -0.57 | 0.53 | -0.60 | 0.51 | -0.73 | 0.48 |
| HSM2 | -0.01 | 0.89 | 0.13 | 0.88 | -0.01 | 1.09 | 0.13 | 1.09 | -0.12 | 1.00 |
| HSM3 | -0.32 | 0.92 | 0.56 | 1.15 | -0.18 | 0.99 | -0.23 | 0.85 | 0.29 | 0.76 |
| HSM4 | 0.32 | 0.77 | -0.24 | 0.94 | -0.56 | 1.07 | 0.46 | 0.86 | 0.44 | 0.74 |
| HSM5 | -0.36 | 0.94 | -0.08 | 0.95 | -0.17 | 0.96 | 0.57 | 0.98 | 0.48 | 0.89 |
| HSM6 | -0.01 | 1.00 | 0.32 | 1.03 | -0.17 | 0.91 | 0.84 | 1.05 | -0.33 | 0.79 |
| HSM7 | -0.14 | 0.88 | -0.29 | 0.93 | 0.44 | 0.92 | 0.43 | 1.35 | -0.45 | 0.79 |
| HSM8 | 0.06 | 0.95 | -0.24 | 0.96 | 0.15 | 1.01 | 0.55 | 1.03 | -0.33 | 0.89 |
| HSM9 | 0.34 | 0.95 | -0.23 | 1.03 | -0.07 | 1.08 | -0.46 | 0.86 | 0.09 | 0.82 |
| HSM10 | 0.11 | 0.92 | -0.21 | 1.08 | -0.28 | 0.96 | -0.23 | 1.11 | 0.54 | 0.77 |

Table shows mean and SD for HSMs for individual cohorts (values represent Z score units from the combined model across cohorts). Differences in mean scores for HSM1 between cohorts, and the artifactually low HSM1 SD score, result from differences in pixel spacing between DXA scanners, leading to distinct aspect ratios.

**Supplementary Table 2 Demographics of the cohorts used in the hip shape GWAS**

| **Study** | **N.total** | **N.GWAS** | **Gender (n)** | **Age (SD)** |
| --- | --- | --- | --- | --- |
| Avon Longitudinal Study of Parents and Children (ALSPAC) | 4,603 | 3,111 | Female | 47.9 (4.3) |
| Framingham Osteoporosis Study  (FOS) | 3,088 | 2,606 | Mixed,  Males (1,042)  Females (1,564) | 63.3 (11.0) |
| Osteoporotic Fractures in Men  (MrOS) | 5,924 | 4,535 | Males | 74.0 (6.0) |
| Study of Osteoporotic Fractures  (SOF) | 1,715 | 1,635 | Females | 72.8 (4.6) |
| TwinsUK | 4,049 | 4,407 | Mixed,  Males (281),  Females (3,766) | 52.5 (13.5) |
| **Total** | 19,379 | 15,934 | Males (5,858)  Females (10,076) |  |

Number of individuals analysed for hip shape (N.total), number of individuals included in the GWAS (N.GWAS), gender and age of participants.

**Supplementary Table 3 Suggestive genome wide significant associations (P<5x10^-9^) from the hip shape GWAS**

| CHROM | BP | SNP | LOCUS | EA | EAF | HSM | BETA | SE | P |
| --- | --- | --- | --- | --- | --- | --- | --- | --- | --- |
| 6 | 45408440 | rs6458443 | *RUNX2* (intronic) | T | 0.360 | 1 | -0.069 | 0.012 | 6.69x10^-9^ |
| 16 | 78426686 | rs6564537 | *WWOX* (intronic) | T | 0.085 | 1 | -0.120 | 0.021 | 1.08x10^-8^ |
| 5 | 4292812 | rs17725170 | *IRX1-ADAMTS16* | A | 0.250 | 2 | -0.079 | 0.017 | 5.69x10^-9^ |

Summary of 3 SNPs close to the cut-off for genome-wide significance, describing site (chromosome, base pair (BP)), locus as defined by position relative to neighbouring genes based on regional association plots, effect (i.e. rare) allele (EA), effect allele frequency (EAF), hip shape mode (HSM) associated with SNP, effect estimate (beta) and genome wide significance P value (P).

**Supplementary Table 4 Genetic associations in height-adjusted GWAS**

|  |  |  |  |  | Non-height adjusted model | | | Height adjusted model | | |
| --- | --- | --- | --- | --- | --- | --- | --- | --- | --- | --- |
| HSM | SNP | Gene/locus | EA |  | BETA | SE | P | BETA | SE | P |
| 1 | rs2158915 | 17q24.3 | G |  | -0.13 | 0.012 | 8.47x10^-27^ | -0.13 | 0.012 | 4.08x10^-27^ |
| 1 | rs1243579 | 14q32.13 | G |  | 0.12 | 0.015 | 2.85x10^-14^ | 0.11 | 0.016 | 7.20x10^-14^ |
| 1 | rs10743612 | 12p11.22 | A |  | 0.093 | 0.013 | 2.91x10^-12^ | 0.092 | 0.013 | 7.04x10^-12^ |
| 1 | rs73197346 | 21q22.12 | C |  | -0.11 | 0.017 | 2.52x10^-10^ | -0.11 | 0.017 | 9.02x10^-11^ |
| 1 | rs59341143 | 4p15.33 | C |  | 0.098 | 0.016 | 6.53x10^-10^ | 0.010 | 0.020 | 4.24x10^-10^ |
| 2 | rs1966265 | *FGFR4* | T |  | 0.13 | 0.014 | 3.73x10^-20^ | 0.12 | 0.014 | 4.01x10^-19^ |
| 2 | rs6537291 | 4q31.21 | A |  | -0.073 | 0.012 | 1.01x10^-9^ | -0.075 | 0.012 | 4.41x10^-10^ |
| 2 | rs1885245 | *ASTN2* | G |  | 0.071 | 0.012 | 4.95x10^-9^ | 0.069 | 0.012 | 1.06x10^-8^ |

Genome-wide significant associations from HSM1 and HSM2 GWAS meta-analyses, with additional adjustment for height.

**Supplementary Table 5 Putative functional SNPs**

| Lead GWAS SNP | Lead functional SNP (RegulomeDB) | RegulomeDB prediction | haploreg annotation | GERP++ | CADD |
| --- | --- | --- | --- | --- | --- |
| rs2158915 | rs9909958  (d=8,808, r^2^=0.963) | Likely (2a);  FOS,STAT3 (ChIP-seq); AP-2 motif | Enhancer^1,2^  Primary DNase^3^ | unconstrained  (-6.7) | neutral  (2.056) |
| rs1243579 | rs12436596  (d=2,715, r^2^=1) | Less likely (3a);  FOS,STAT3 (ChIP-seq); PU.1 motif | Enhancer^2^’^3^ | **constrained  (4.05)** | **pathogenic  (19.09)** |
| rs10743612 | rs11049197  (d=18,659, r^2^=0.647) | Likely (2b); POLR2A,CCNT2,E2F6, MAX, MYC, EP300, GATA2, MYC, TEAD4 ,BHLHE40, FOXA1,JUND,NRF2, RCOR1 (ChIP-seq); RREB-1 motif | Primary DNase^1,^ Enhancer^1,2,3^ Promoter^1,2,3^ | unconstrained  (-4.61) | neutral  (2.871) |
| rs59341143 | rs10034452  (d=834, r^2^ = 1) | Likely (2b); EZH2 (ChIP-seq); Elf,Ets, HNF motifs | Enhancer^3^ | unconstrained  (-0.486) | neutral  (6.891) |
| rs1966265 | rs12519145  (d=38,892, r^2^=0.775) | Highly Likely (1f); YY1, ZNF263 (ChIP-seq); RAB24 eQTL (lymphoblastoid) | Primary DNase^2,3^ | unconstrained  (-0.176) | neutral  (3.358) |
| rs6537291 | rs12509311  (d=14,588, r^2^=0.967) | Less Likely (3a); CTCF,RAD21 (ChIP-seq); Oct-1, MEF-2 motif | None | unconstrained  (-1.67) | neutral  (7.294) |
| rs1885245 | rs1895062  (d=78,927, r^2^=0.929) | Likely (2b);  CEBPB, FOS, STAT3, ARID3A, EP300, FOS, FOSL2, JUNB, JUND, JUN, MAFF, RCOR1, TEAD4, USF2(ChIP-seq);  IPF1, Oct-1, POUF3F2 motifs | Enhancer ^1,2,3^ | **constrained**  (2.72) | neutral  (12.7) |

SNPs in LD (r^2^>0.6) with the lead GWAS SNP were evaluated in RegulomeDB, and SNPs predicted to have functional consequences further evaluated by Haploreg, GERP++ and CADD. Distance from lead SNP in base pairs (d), r^2^ with lead SNP (r^2^). Haploreg annotation: Regulatory chromatin states from DNAse and histone ChIP-Seq (Roadmap Epigenomics Consortium, 2015) for osteoblast primary cells ^1^, bone marrow derived cultured mesenchymal stem cells (chondrocyte progenitor cells) ^2^, and mesenchymal stem cell derived chondrocyte cultured cells ^3^

**Supplementary Table 6 Comparison between SNPs associated in previous** Statistical Shape Modeling (SSM**) studies and associations from this SSM study.**

| SNP | gene | study | modes associated | study.p | HSM1.P | HSM2.P |
| --- | --- | --- | --- | --- | --- | --- |
| rs5009270 | *IFRD1* | Lindner | 5,7,10 | 0.006 | 0.14 | 0.04 |
| rs4836732 | *ASTN2* | Lindner | 3,4,9 | 0.0004 | 0.75 | 3.45E-06 |
| rs6976 | *GLT8D1* | Lindner | 5,8,12 | 0.003 | 0.58 | 0.12 |
| rs1516893 | *TMEM38B* | Lindner | 7,10,11 | 0.004 | 0.60 | 0.57 |
| rs12901499 | *SMAD3* | Lindner | 6,7,9 | 0.002 | 0.32 | 0.20 |
| rs288326 | *FRZB* | Baker-Lepain | 2 | 0.02 | 0.85 | 0.44 |
| rs7775 | *FRZB* | Baker-Lepain | 2 | 0.02 | 0.44 | 0.21 |

For the modes associated in the original studies, Lindner used a multivariate model which assessed the combined association of the top three contributing modes as assessed by canonical correlation analysis, whereas Baker-Lepain performed a univariate analysis on single modes. P-value reported for the Lindner or Baker-Lepain study (study.P). P-value for HSM (HSM1.P) and HSM2 (HSM2.P) in our hip shape meta-analysis GWAS.

**Supplementary Figure 1: Hip shape modes 1-10**

**
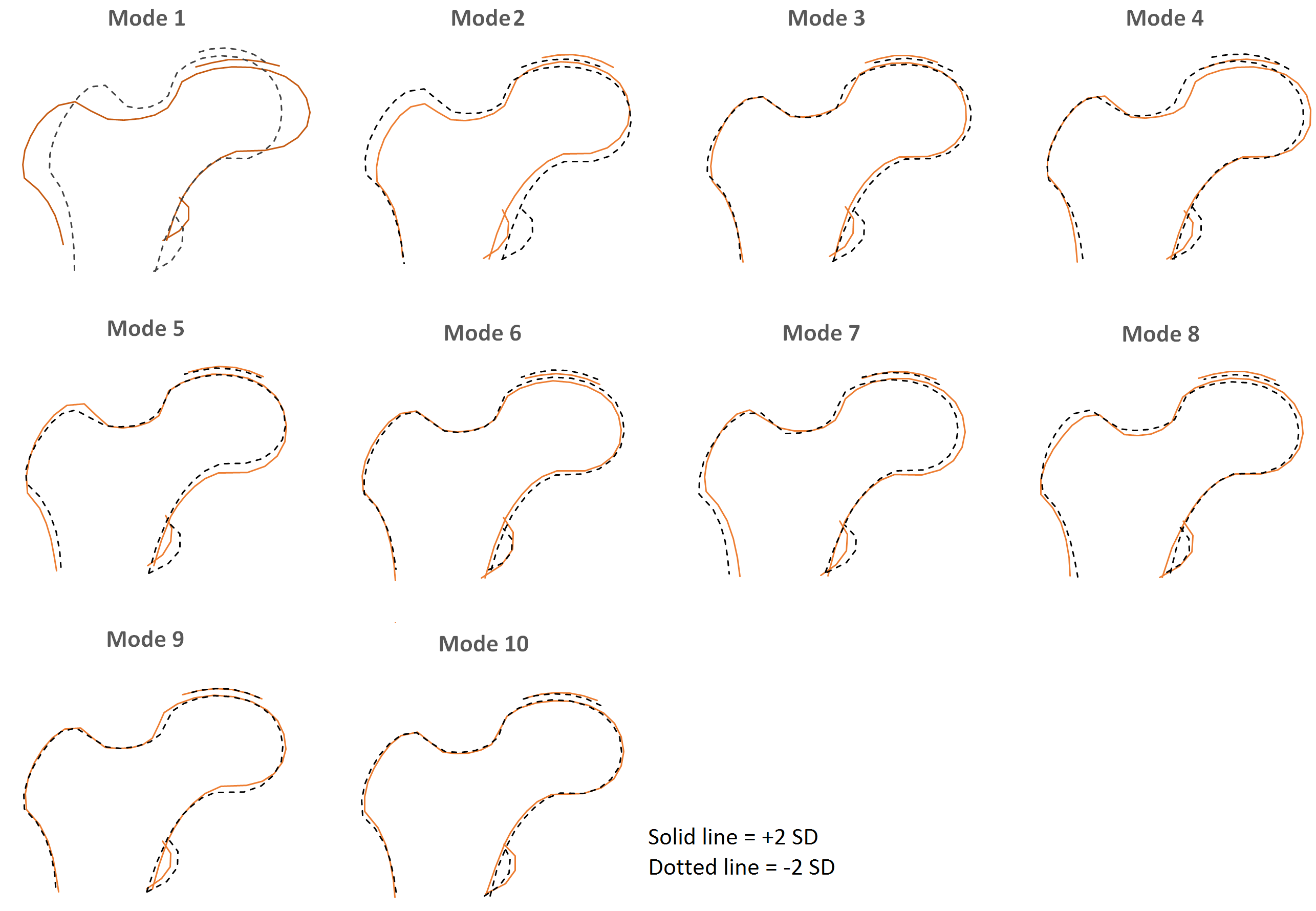
**

**Supplementary Figure 2: Meta-analysis QQ plots for each of the first ten hip shape modes**


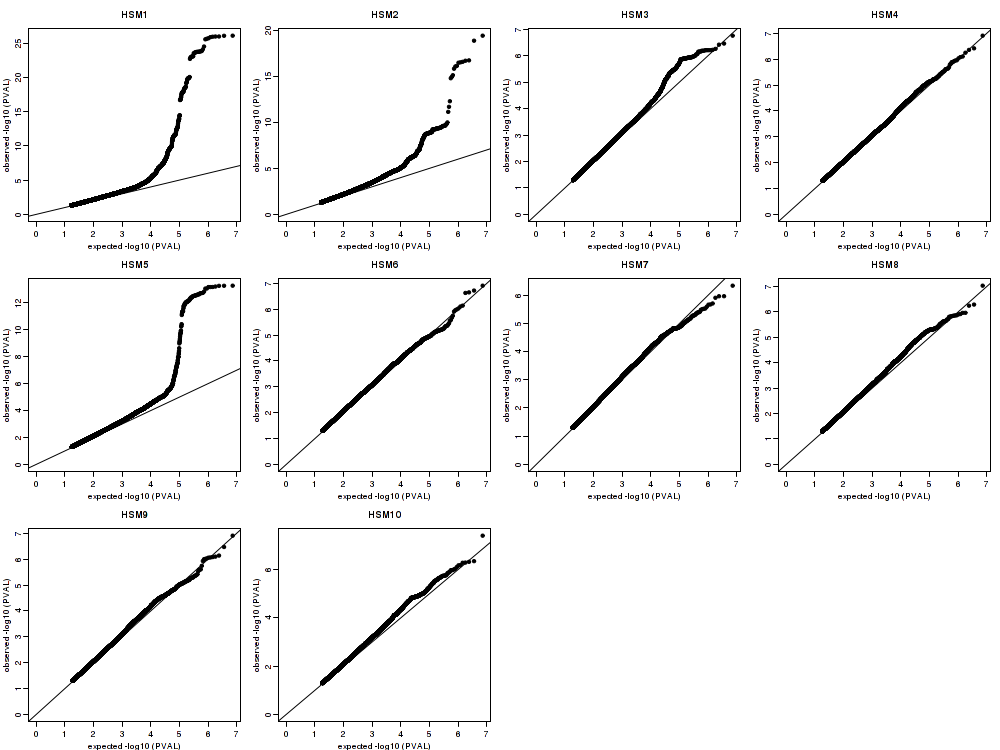


**Supplementary Figure 3: Meta-analysis Manhattan plots**

HSM1


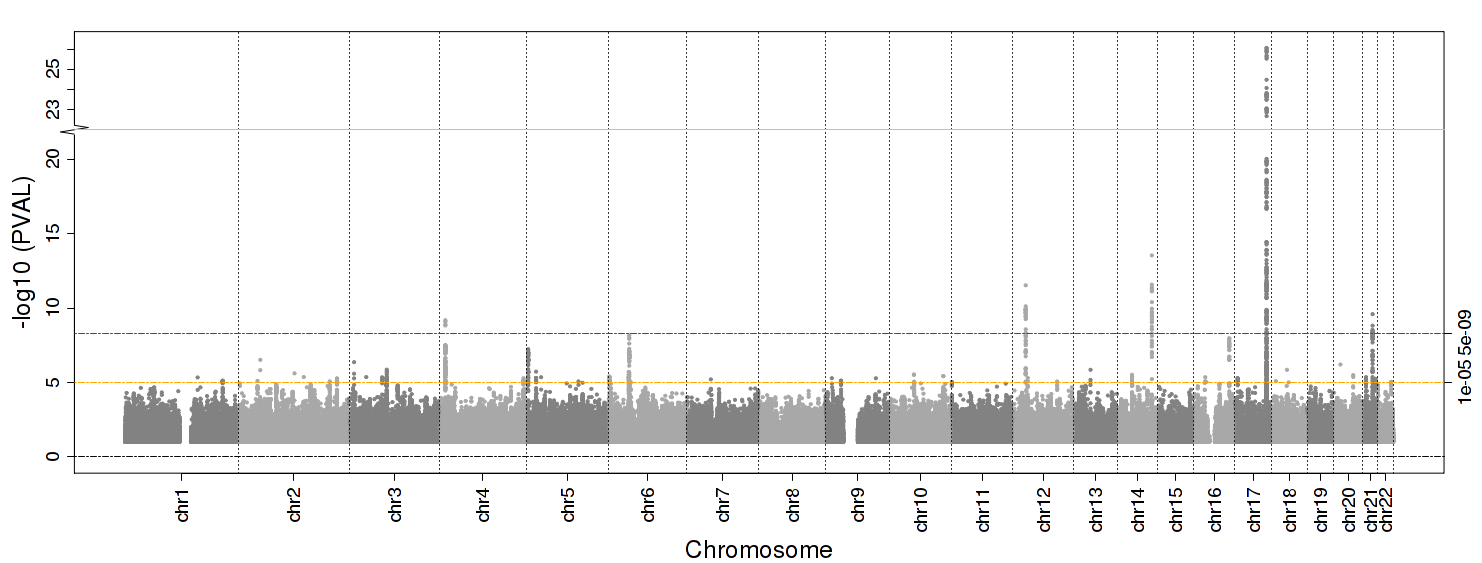


HSM2


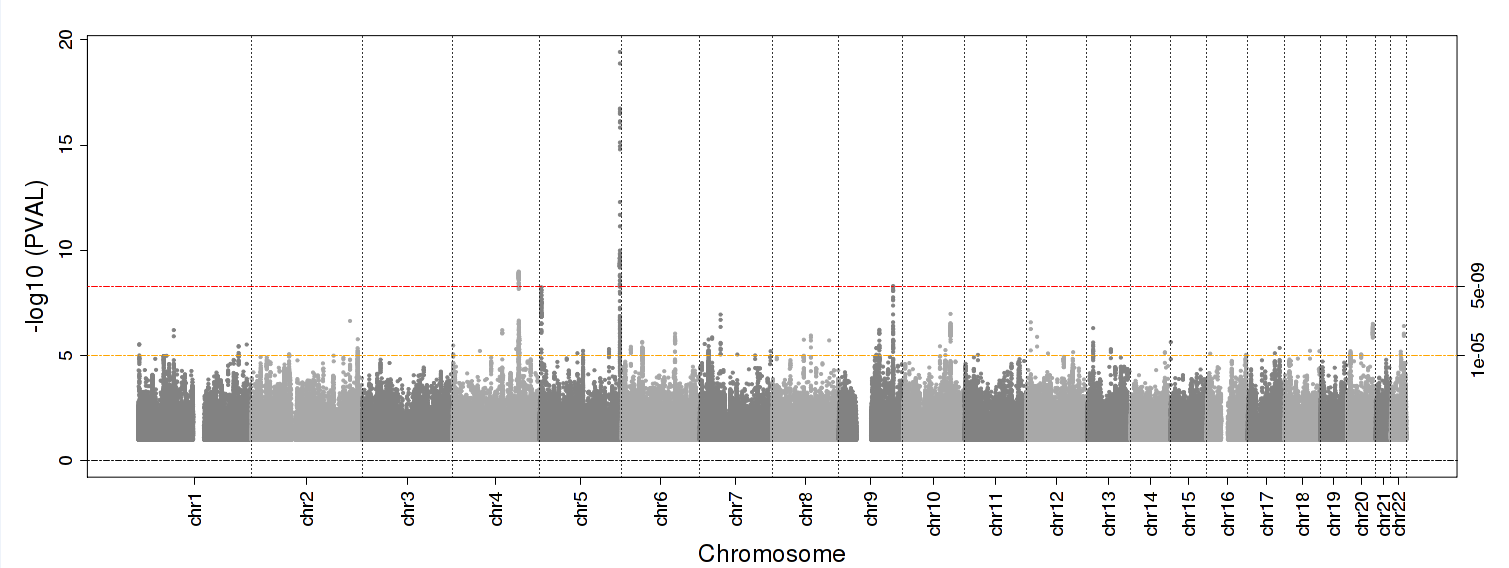


HSM5


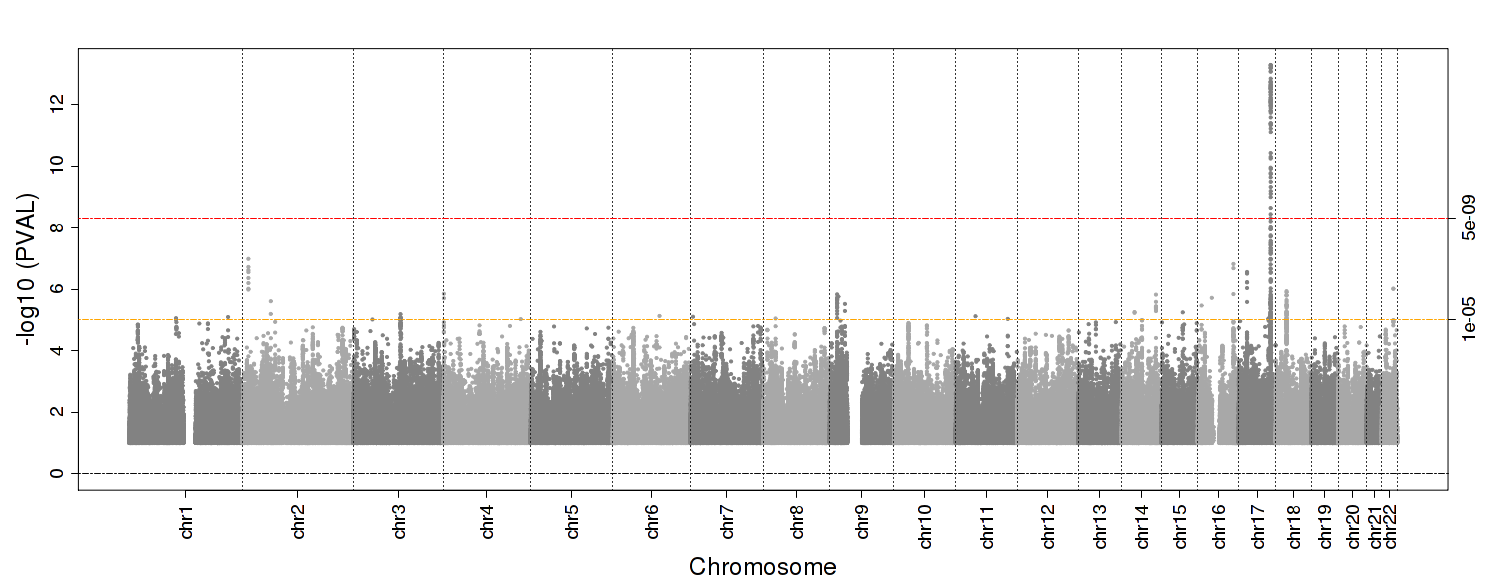


**Supplementary Figure 4 Regional association plot for rs2158915 locus associations with hip shape**

1. HSM 1


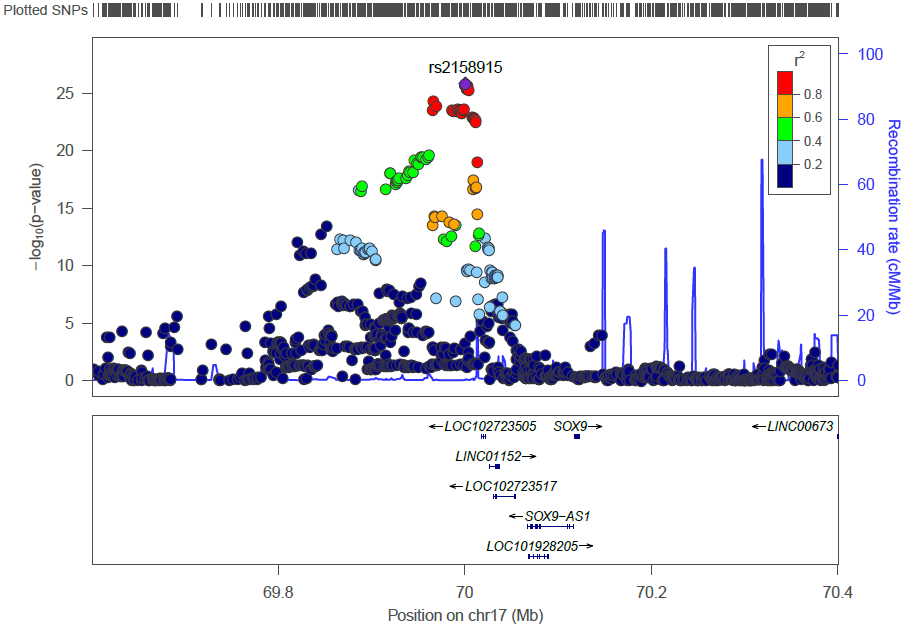


1. HSM5


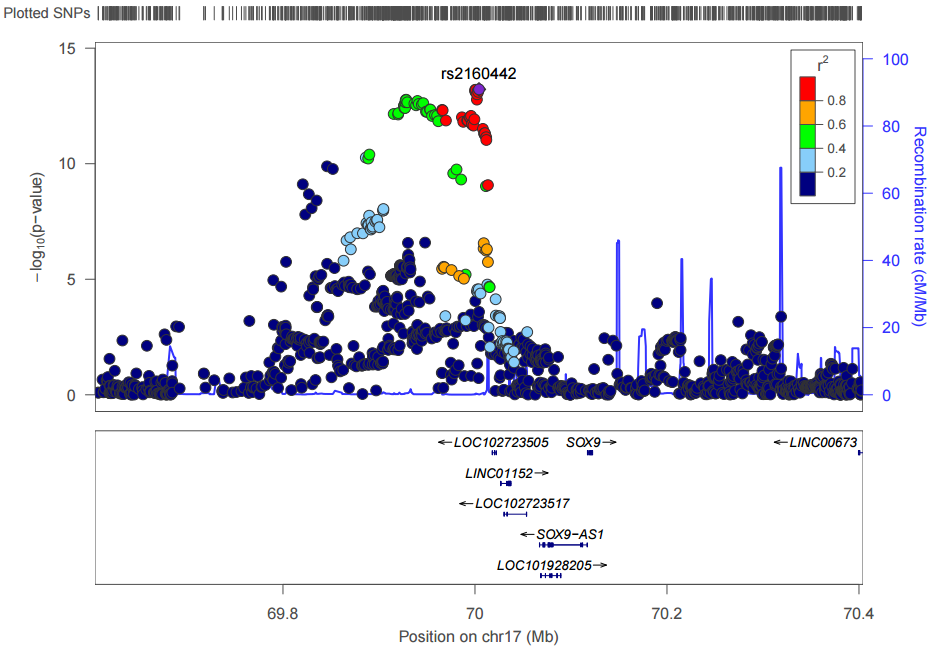


**Supplementary Figure 5 Regional association plot for rs1243579 association with HSM1**

**
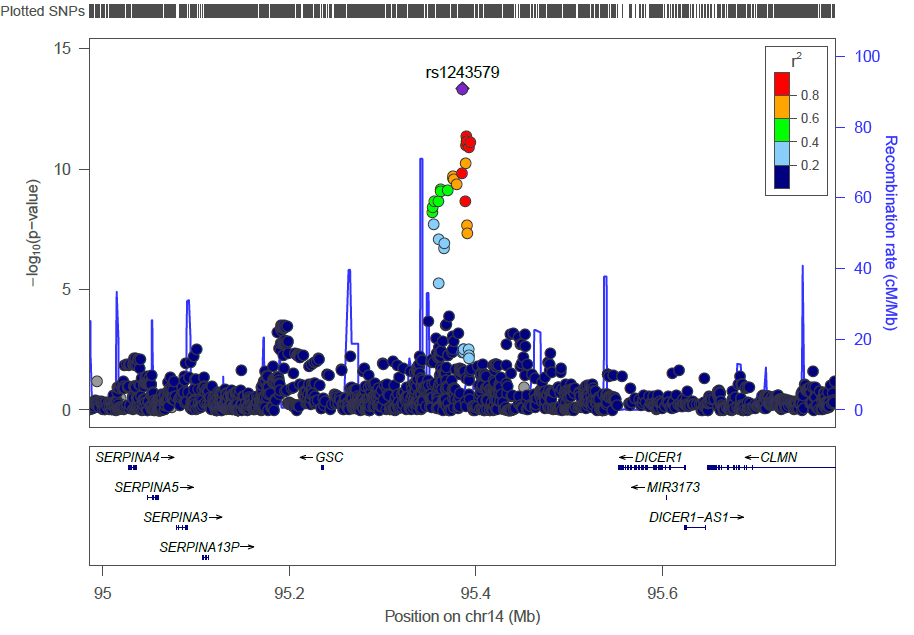
**

**Supplementary Figure 6 Regional association plot for rs10743612 association with HSM1**

**
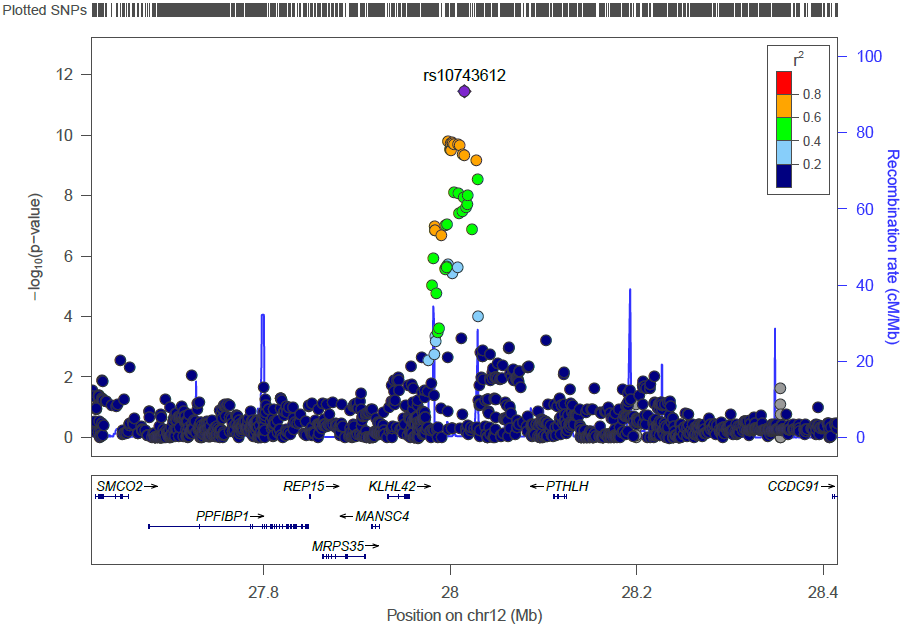
**


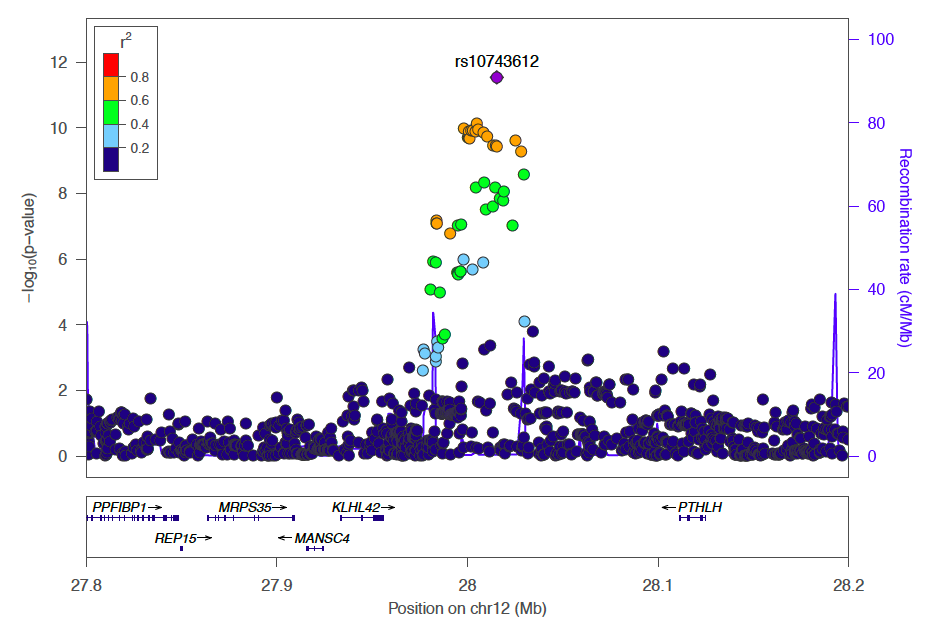


**rs10492367**

**Supplementary Figure 7 Regional association plot for rs73197346 association with HSM1**

**
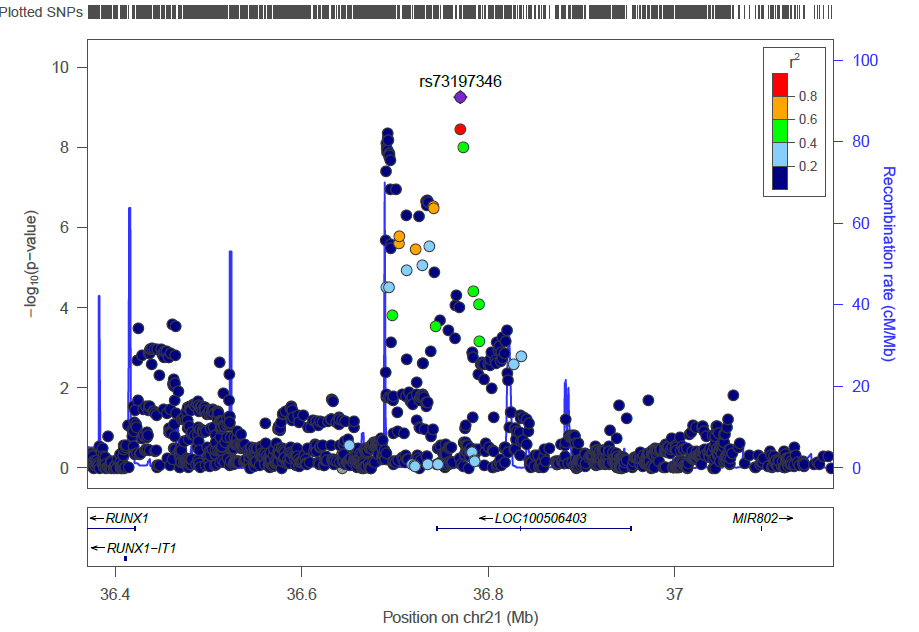
**

**Supplementary Figure 8 Regional association plot for rs59341143 association with HSM1**


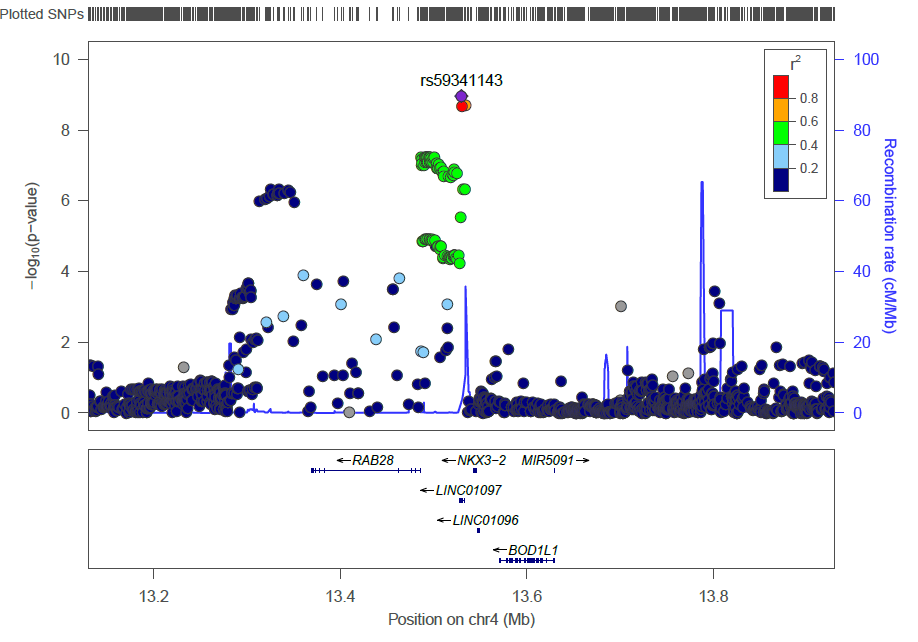


S**upplementary Figure 9 Regional association plot for rs1966265 association with HSM2**


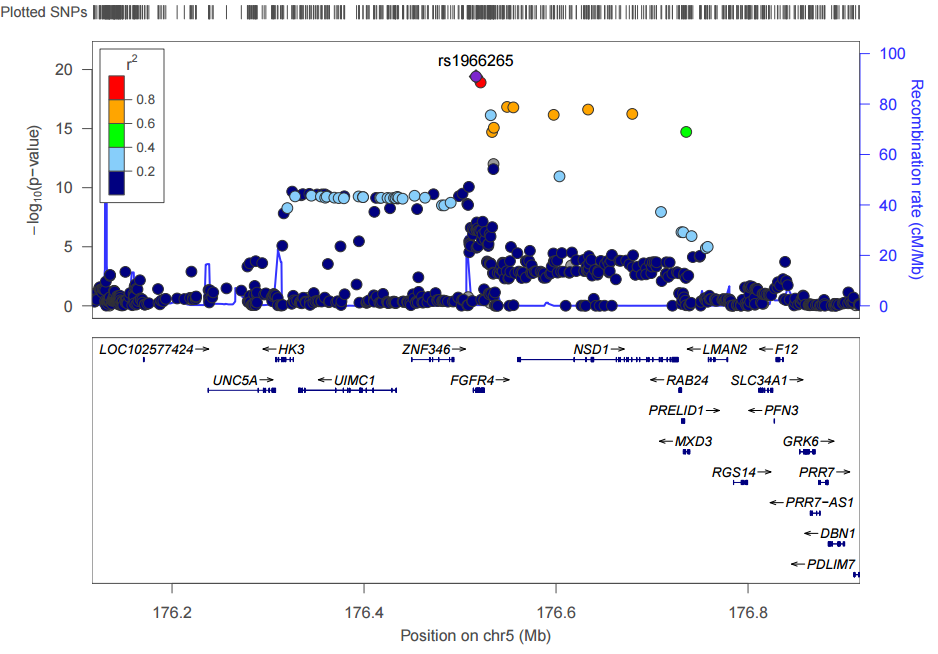


**Supplementary Figure 10 Regional association plot for rs6537291 association with HSM2**


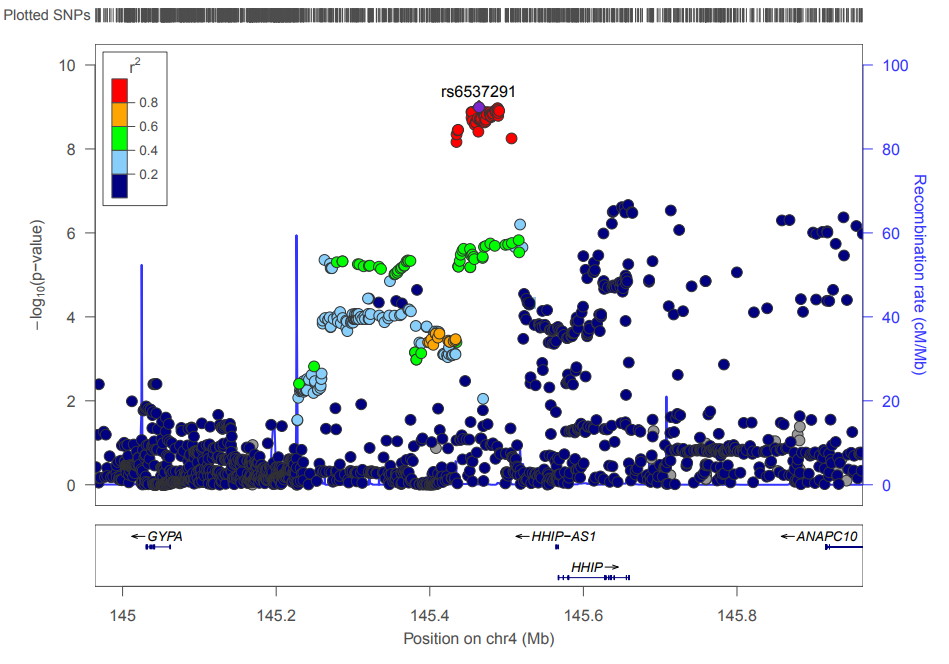


**Supplementary Figure 11 Regional association plot for rs1885245 association with HSM2**


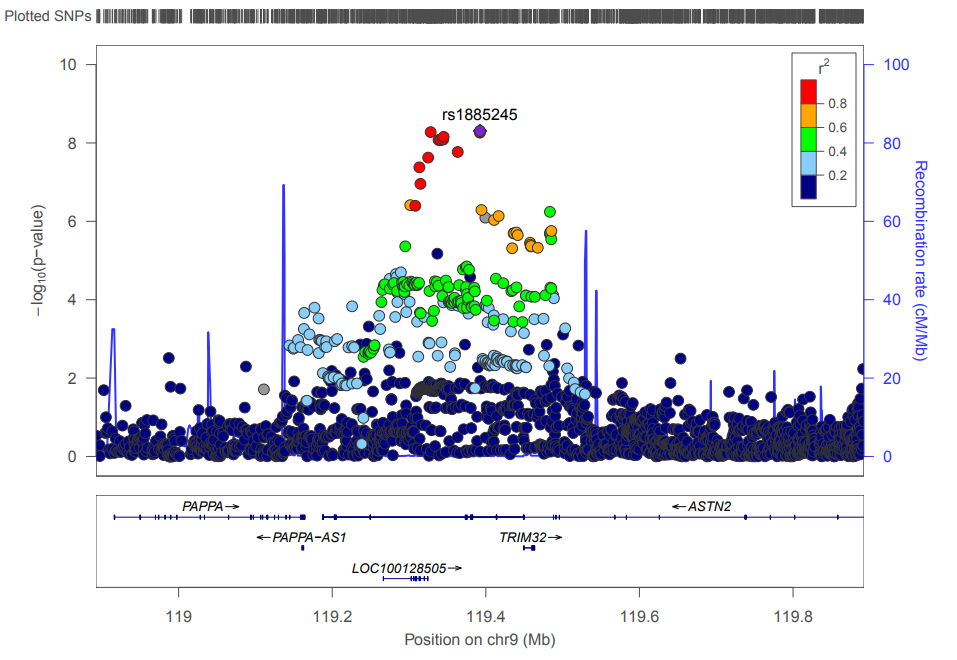


**Supplementary Figure 12 Lead variant loci intersecting regions of open chromatin during proximal femur development**


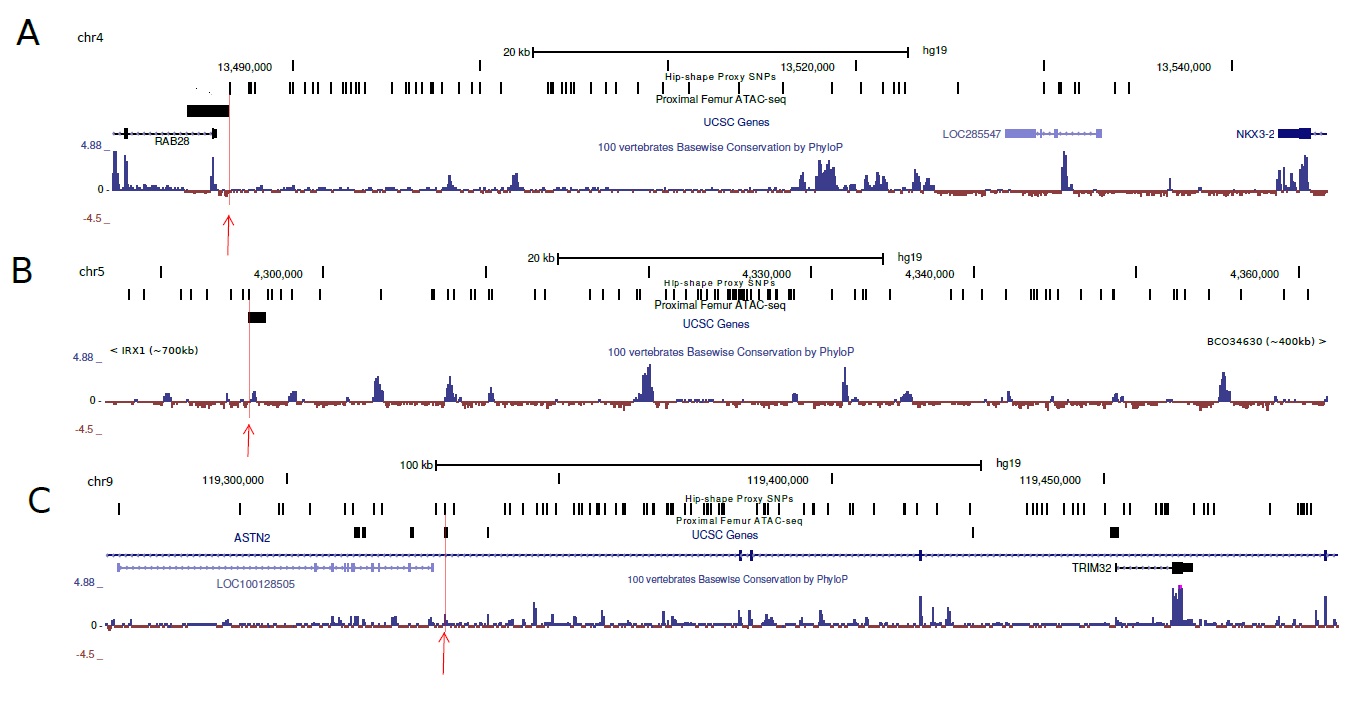

Supplement: Supplementary file 2 — Supporting Data S1. [file JBMR-34-241-s002.docx]
